# mTORC1 is Required for Brown Adipose Tissue Recruitment and Metabolic Adaptation to Cold

**DOI:** 10.1038/srep37223

**Published:** 2016-11-23

**Authors:** Sébastien M. Labbé, Mathilde Mouchiroud, Alexandre Caron, Blandine Secco, Elizaveta Freinkman, Guillaume Lamoureux, Yves Gélinas, Roger Lecomte, Yohan Bossé, Patricia Chimin, William T. Festuccia, Denis Richard, Mathieu Laplante

**Affiliations:** 1 Institut universitaire de cardiologie et de pneumologie de Québec - Université Laval, 2725 chemin Sainte-Foy, Québec, QC, G1V 4G5, Canada; 2 Département de Médecine, Faculté de Médecine, Université Laval, Québec, QC, Canada; 3 Whitehead Institute for Biomedical Research, 9 Cambridge Center, Cambridge, MA 02142, USA; 4 Centre d’imagerie moléculaire de Sherbrooke (CIMS), Département de Médecine nucléaire et radiobiologie, Université de Sherbrooke, Sherbrooke, J1H 5N4, Canada; 5 Department of Physiology & Biophysics, Institute of Biomedical Sciences, University of São Paulo, São Paulo, 05508-000, Brazil

## Abstract

In response to cold, brown adipose tissue (BAT) increases its metabolic rate and expands its mass to produce heat required for survival, a process known as BAT recruitment. The mechanistic target of rapamycin complex 1 (mTORC1) controls metabolism, cell growth and proliferation, but its role in regulating BAT recruitment in response to chronic cold stimulation is unknown. Here, we show that cold activates mTORC1 in BAT, an effect that depends on the sympathetic nervous system. Adipocyte-specific mTORC1 loss in mice completely blocks cold-induced BAT expansion and severely impairs mitochondrial biogenesis. Accordingly, mTORC1 loss reduces oxygen consumption and causes a severe defect in BAT oxidative metabolism upon cold exposure. Using *in vivo* metabolic imaging, metabolomics and transcriptomics, we show that mTORC1 deletion impairs glucose and lipid oxidation, an effect linked to a defect in tricarboxylic acid (TCA) cycle activity. These analyses also reveal a severe defect in nucleotide synthesis in the absence of mTORC1. Overall, these findings demonstrate an essential role for mTORC1 in the regulation of BAT recruitment and metabolism in response to cold.

In mammals, brown adipose tissue (BAT) serves as a key heat-producing organ required to maintain body temperature and homeothermy^1^. In response to cold, activation of the sympathetic nervous system (SNS) rapidly turns on lipolysis and thermogenesis along with a specific transcriptional program required for heat production in brown adipocytes^1^,^2^. BAT thermogenesis depends on the uncoupling protein 1 (UCP1), a mitochondrial transporter protein that deviates protons across the inner mitochondrial membrane, uncoupling oxidative phosphorylation from ATP synthesis^1^,^3^. As a result, energy is dissipated in the form of heat, which facilitates the adaptation to a cold environment.

Until recently, BAT was thought to be present and active only in small mammals and human newborns^4^,^5^. With the use of positron emission tomography (PET), several groups confirmed over the last years the existence of BAT in adult humans^6^,^7^,^8^,^9^. BAT can be found in supraclavicular, cervical, paraspinal and mediastinal regions^8^,^10^,^11^ acting as a heat-producing organ in adult humans^9^. As demonstrated in rodents, cold exposure rapidly activates the uptake of glucose, non-esterified fatty acids (NEFAs) and triglyceride (TG)-derived fatty acids to support oxidative metabolism and heat production in BAT^12^,^13^,^14^. These results suggest that BAT may be exploited to dissipate energy excess, fight obesity and improve glucose and lipid homeostasis in humans^13^,^15^,^16^,^17^.

In response to chronic adrenergic stimulation, BAT expands its mass through a combination of hyperplasia and hypertrophy and increases mitochondrial number to support thermogenesis, a phenomenon observed in both rodents^1^,^18^,^19^,^20^,^21^,^22^ and humans^23^,^24^. BAT expansion has been associated with improved insulin sensitivity in patients with type 2 diabetes^15^,^24^. The signaling events that contribute to BAT expansion during chronic cold exposure are not well characterized. The mechanistic target of rapamycin (mTOR) complex 1 (mTORC1) is a central regulator of cell growth and metabolism^25^. In response to nutrients and growth factors, mTORC1 activates several anabolic processes including protein and lipid synthesis. Here, we show that mTORC1 activity is robustly increased in BAT of mice exposed to cold. Interestingly, we report that BAT denervation is sufficient to abrogate the elevation in mTORC1 activity, indicating that cold activates mTORC1 through the SNS. Furthermore, adipose-specific loss of Raptor, an essential component of mTORC1, completely blocks cold-induced BAT mass expansion, reduces mitochondrial biogenesis and severely impairs BAT oxidative metabolism. Using a combination of *in vivo* metabolic imaging, metabolomics and transcriptomics, we show that mTORC1 loss impairs glucose and lipid oxidation, an effect linked to a defect in TCA cycle activity. These analyses also reveal a defect in nucleotide synthesis that may contribute to impair brown adipocyte proliferation. Our findings demonstrate an essential role for mTORC1 in the regulation of BAT recruitment and metabolism in response to cold.

## Results

### Cold exposure promotes mTORC1 activity in BAT

**Figure 1:**
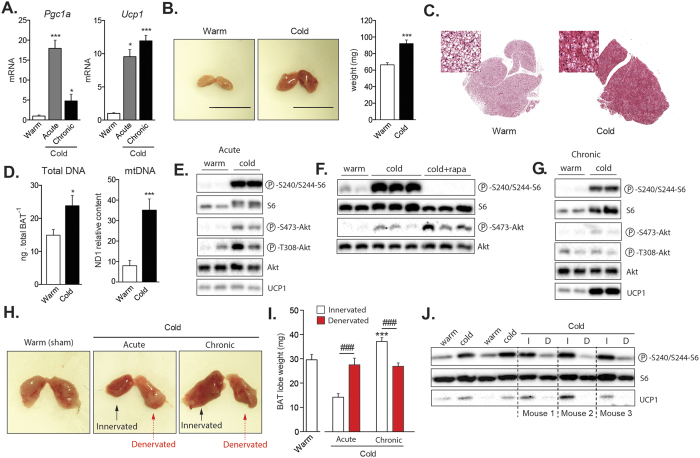
Cold exposure promotes mTORC1 activity in BAT through the sympathetic nervous system. Mice were either kept at thermoneutrality (warm; 30 °C) or exposed to cold (10 °C) during 2 weeks. Acute cold exposure (6-hour at 10 °C) was performed following 2 weeks at thermoneutrality. (**A**) Gene expression of *Pgc1a* and *Ucp1* in BAT (n = 8 for warm; n = 5 for acute cold; n = 8 for chronic cold; mean +/− SEM; ANOVA; *P < 0.05; ***P < 0.001). (**B**) (Left) Representative pictures of BAT collected from mice kept at thermoneutrality or exposed to cold for 2 weeks. (Right) BAT weight measured from the same animals (n = 14–22; mean +/− SEM; t-test; ***P < 0.001). (**C**) Representative H&E staining of representative BAT collected from mice kept at thermoneutrality or in cold for 2 weeks. (**D**) (Left) Total DNA content (n = 7–8; mean +/− SEM; t-test; *P < 0.05) and (right) mtDNA content measured in mice kept in warm or cold for 2 weeks. (n = 8; mean +/− SEM; t-test; ***P < 0.05). (**E**) Western blots performed on BAT collected from mice following warm or acute cold exposure. Mice were fasted during the cold exposure (6-hour period). (**F**) Western blots performed on BAT collected from mice following warm or acute cold exposure. A group of cold-exposed mice received an acute intraperitoneal injection of rapamycin (2 mg/kg) one-hour prior to the cold exposure (6-hour period). (**G**) Western blots performed on BAT collected from mice following warm or chronic cold exposure. Mice were fasted during the last 6 hours. (**H**) Representative pictures of unilateral BAT denervation. (Left) Sham mice kept at thermoneutrality, (middle) unilateral BAT denervation following acute cold or (right) chronic cold exposure. (**I**) BAT lobe weight following unilateral BAT denervation (n = 8 for Sham-Warm; n = 5 for acute cold; n = 10 for chronic cold; mean +/− SEM; Two-way ANOVA; ***P < 0.001 vs. Sham-Warm lobe; ^###^P < 0.001 vs. Innervated lobe). (**J**) Western blots performed on BAT collected from mice with unilateral BAT denervation. Mice were fasted during the last 6 hours. Each mouse was its own control. In this panel, (**I**) refers to the innervated lobe and and (**D**) refers to the denervated lobe. All gels have been run under the same experimental conditions.

Cold exposure rapidly activates thermogenesis in BAT^1^,^26^. The rapid induction of BAT thermogenic capacity is highly dependent on peroxisome proliferator-activated receptor gamma coactivator 1-alpha (PGC-1α), a key transcriptional regulator that promotes the expression of several thermogenic genes, including *Ucp1*^27^. Confirming previous findings, we observed that both acute (6 hours) and chronic (2 weeks) cold exposure (10 °C) strongly enhance the expression of *Pgc1a* and *Ucp1* in BAT (Fig. 1A). Other genes known to participate in the metabolic adaptation to cold were also induced in these conditions (Figure S1A).

As previously reported, we observed that chronic exposure to cold induced the expansion of BAT, a process required for the long-term adaptation to cold^1^,^22^ (Fig. 1B). Histological and biochemical analyses revealed that chronic cold exposure increased BAT density, total DNA and mitochondrial DNA (mtDNA) content, indicating that the elevation in BAT mass is the result of increased cell number and mitochondrial mass (Fig. 1C,D). Because mTORC1 plays a crucial role in regulating anabolic processes required for cell growth, cell proliferation and mitochondrial biogenesis, we hypothesized that this signaling node may be particularly important for the adaptation of BAT to cold temperature. To specifically test the impact of cold on mTORC1 signaling, mice were acutely or chronically exposed to cold and BAT was collected for analyses. As shown before, we observed that cold exposure rapidly promotes food intake in mice (Figure S1B). Because food intake elevates circulating levels of insulin and nutrients, two well-characterized upstream activators of mTORC1, all the mice were fasted for 6 hours before sacrifice to avoid any contribution of these factors to the modulation of mTOR signaling. As shown in Fig. 1E, we observed that acute cold exposure induced a robust increase in the phosphorylation levels of ribosomal protein S6, a readout of mTORC1 activity. These results confirm the findings of two recent reports showing that adrenergic stimulation and cold both activate mTORC1 in WAT and BAT^28^,^29^. Interestingly, pre-treating mice with the mTORC1 inhibitor rapamycin was sufficient to block cold-induced phosphorylation of S6 (Fig. 1F). To test whether the elevation in mTOR signaling was sustained over time, mice were exposed to cold for two weeks. We observed that mTORC1 activity was also highly induced in this condition (Fig. 1G). Altogether, these results indicate that both acute and chronic cold exposure activate mTORC1 in BAT. Interestingly, the activation of mTORC1 by cold was associated with an elevation in the phosphorylation of protein kinase B/Akt (Akt) (Fig. 1E to G), indicating that the phosphoinositide-3 kinase (PI3K)/mTORC2 axis was also activated by cold, as recently suggested^30^.

### Cold-induced mTORC1 activation in BAT depends on the sympathetic nervous system

The elevation in mTORC1 activity observed in response to cold is robust. Such rise is also surprising considering that circulating insulin, a key activator of mTORC1, was not increased in cold-exposed fasted animals. In fact, a reduction in plasma insulin was even observed in mice exposed to cold (Figure S1C). These results strongly support the idea that other factors, not related to the nutritional status, contribute to activate mTORC1 in response to cold. It has long been known that BAT thermogenesis is regulated by the central nervous system through the SNS^31^. To test the contribution of the SNS to the activation of mTORC1, we first injected mice with CL316,243 (CL), a highly selective β3-adrenergic agonist that activates BAT thermogenesis^32^,^33^,^34^. We observed that CL strongly increased mTORC1 activity in BAT (Figure S1D). However, we also found that CL induced a robust increase in circulating insulin levels following injection (Figure S1E), an effect that has been reported by others^35^. Because insulin activates mTORC1, a direct role for the SNS in cold-induced mTORC1 activation cannot be demonstrated using this approach. To circumvent this limitation, we performed unilateral BAT denervation and acutely or chronically exposed mice to cold. This approach is extremely powerful as it allows a direct comparison between the innervated and the denervated lobes of BAT within the same mouse, thus ruling out the contribution of humoral factors to the regulation of the pathway. Confirming the validity of the surgical procedure, we observed that blocking the adrenergic drive to BAT reduced glucose uptake, impaired the mobilization of TG and reduced mtDNA content (Figure S1F to S1H). Unilateral BAT denervation completely prevented the expansion of the denervated lobe normally observed in response to chronic cold exposure (Fig. 1H,I). Strikingly, we found that this procedure also prevented the induction of mTORC1 activity observed in response to cold, indicating a key role for the SNS in this effect (Fig. 1J).

### Loss of mTORC1 reduces mitochondrial biogenesis and prevents BAT expansion in response to cold

**Figure 2:**
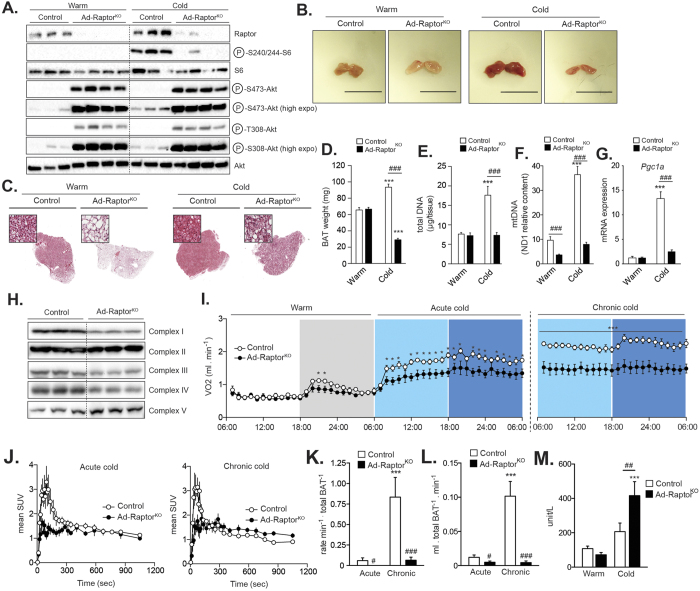
Loss of mTORC1 impairs cold-induced BAT expansion, mitochondrial biogenesis, and oxidative metabolism. Control and Ad-Raptor^KO^ mice were either kept at thermoneutrality (warm; 30 °C) or exposed to cold (10 °C) for 2 weeks. (**A**) Western blots performed on BAT following warm or acute cold exposure. All gels have been run under the same experimental conditions. (**B**) Representative pictures of BAT and (**C**) H&E staining of representative BAT collected after warm or chronic cold exposure. (**D**) Total BAT mass (n = 13–17; mean +/− SEM; Two-way ANOVA; ***P < 0.001 vs. Warm; ^###^P < 0.001 vs. Control). (**E**) Total DNA content (n = 7–9; mean +/− SEM; Two-way ANOVA; ***P < 0.001 vs. Warm; ^###^P < 0.001 vs. Control). (**F**) mtDNA content (n = 7–9; mean +/− SEM; Two-way ANOVA; ***P < 0.001 vs. Warm; ^###^P < 0.001 vs. Control). (**G**) Gene expression of *Pgc1a* (n = 4; mean +/− SEM; Two-way ANOVA; ***P < 0.001 vs. Warm; ^###^P < 0.001 vs. Control). (**H**) Western blots performed on BAT collected from control and Ad-Raptor^KO^ mice following chronic cold exposure. All gels were run under the same experimental conditions. (**I**) Oxygen consumption at thermoneutrality (warm) and during the transition to cold. (n = 6–8; mean +/− SEM; t-test; *P < 0.05; ***P < 0.001). White section: thermoneutrality, when the lights were on; Grey section: thermoneutrality, when the lights were off; Light blue section: cold, when the lights were on; Dark blue section: cold, when the lights were off. (**J**) Mean SUV time-activity curves of ^11^C-acetate during (left) acute and (right) chronic cold exposure (n = 4–5; mean +/− SEM). (**K**) Total BAT oxidative activity index corrected for tissue weight (n = 6–8; mean +/−SEM; Two-way ANOVA; ***P < 0.001 vs. Warm; ^#^P < 0.05 vs. Control; ^###^P < 0.001 vs. Control). (**L**) Total BAT blood flow index corrected for tissue weight (n = 4–5; mean +/− SEM; Two-way ANOVA; ***P < 0.001 vs. Warm; ^#^P < 0.05 vs. Control; ^###^P < 0.001 vs. Control). (**M**) Plasma CK activity (n = 10–13; mean +/− SEM; Two-way ANOVA; ***P < 0.001 vs. Warm; ^###^P < 0.001 vs. Control).

In order to test the importance of mTORC1 in regulating BAT function and expansion in response to cold, we crossed *Raptor*^Lox/Lox^ mice^36^ with mice expressing the Cre-recombinase under the control of the *Adiponectin* promoter^37^. This method was efficient to specifically delete Raptor, an essential component of mTORC1, in adipose tissues (Ad-Raptor^KO^) (Fig. 2A and Figure S2A). At thermoneutrality and following chronic cold exposure, Ad-Raptor^KO^ mice were slightly heavier than control animals, despite a significant reduction in WAT masses (Figure S2B). Consistent with the reduction in WAT size, Ad-Raptor^KO^ mice showed lower levels of circulating leptin and NEFAs (Figure S2C).

As shown in Fig. 2A, the loss of Raptor completely prevented the phosphorylation of S6 induced by acute cold exposure. As previously reported, Raptor deletion caused an important increase in the phosphorylation of Akt (Fig. 2A), an effect linked to the reduction in the negative feedback loops from mTORC1 to PI3K-Akt^36^,^38^,^39^. Morphological, histological and biochemical analyses were next performed in BAT to characterize the physiological consequences linked to chronic loss of mTORC1. We first observed that BAT isolated from Ad-Raptor^KO^ mice housed at thermoneutrality (warm; 30 °C) was whiter than control BAT (Fig. 2B, left panel). Histological analyses revealed the presence of larger lipid droplets accumulated in brown adipocytes of Ad-Raptor^KO^ (Fig. 2C, left panel). Interestingly, the weight of BAT and the total number of cells, as measured by the DNA content, were not different between control and Ad-Raptor^KO^ mice housed in a warm environment (Fig. 2D,E). However, we noticed an important reduction in mtDNA content in BAT of Ad-Raptor^KO^, indicating that mTORC1 is required to support basal mitochondrial biogenesis (Fig. 2F). The impact of Raptor deletion was also tested in animals exposed to cold for two weeks. Strikingly, we found that loss of mTORC1 completely blocked the expansion of BAT observed in response to chronic cold exposure (Fig. 2B, right panel). We even observed a reduction in BAT weight in Ad-Raptor^KO^ mice exposed to cold, an effect likely linked to the reduction in intracellular TG (Fig. 2C,D). Contrary to control mice, Ad-Raptor^KO^ mice did not show any increase in BAT DNA content following cold exposure (Fig. 2E). These animals also failed to efficiently increase mitochondrial biogenesis, as demonstrated by the severe reduction in mtDNA content (Fig. 2F). This latter effect was associated with a profound defect in the expression of *Pgc1a* (Fig. 2G) and *Ucp1* (Figure S2D). We also measured a reduction in the protein levels of complex I, III and IV of the electron transport in response to Raptor deletion (Fig. 2H). Altogether, these results indicate that mTORC1 controls mitochondrial biogenesis and plays a crucial role in promoting BAT expansion in response to cold.

### Loss of mTORC1 impairs BAT oxidative metabolism and non-shivering thermogenesis

To characterize the systemic consequences associated with the loss of mTORC1 in BAT, we performed indirect calorimetry experiments in control and Ad-Raptor^KO^ mice. In a warm environment, a condition in which BAT is not solicited, we did not observe any difference in oxygen consumption between the groups in the light phase (Fig. 2I, white section). We noted that Ad-Raptor^KO^ mice had a tendency for lower oxygen consumption in the dark phase (Fig. 2I, grey section). As reported before^40^, acute and chronic cold exposure sequentially increased oxygen consumption in control mice, an indicator of increased non-shivering thermogenesis activation and chronic adaptation, respectively (Fig. 2I). In response to acute cold, Ad-Raptor^KO^ mice showed lower oxygen consumption compared to control animals (Fig. 2I). Importantly, the difference between control and Ad-Raptor^KO^ mice were amplified in response to chronic cold exposure (Fig. 2I). The inability of Ad-Raptor^KO^ to activate mitochondrial biogenesis and expand BAT mass upon chronic cold exposure likely explains this result.

To determine whether the reduction in oxygen consumption in Ad-Raptor^KO^ was linked to a specific defect in BAT function, we performed positron emission tomography (PET) experiments using ^11^C-acetate, a metabolic tracer that allows live measurement of oxidative metabolism by the tricarboxylic acid (TCA) cycle^9^,^14^,^41^. Time-activity curves calculated from ^11^C-acetate dynamic scans revealed a striking defect in BAT metabolic activity in Ad-Raptor^KO^ mice exposed to cold (Fig. 2J). Mathematical modeling derived from the ^11^C-acetate time-activity curves confirmed that BAT from Ad-Raptor^KO^ mice displayed a profound defect in oxidative metabolism (Fig. 2K). These differences were even amplified when the data were corrected for total BAT mass, which supports the indirect calorimetry data presented above. Importantly, we did not observe any impact of mTORC1 deletion on oxidative metabolism in any other adipose tissues tested, showing that this effect was specific to BAT (Figure S2E-S2F). Three-compartment kinetic modeling has previously demonstrated that ^11^C-acetate can be used as a reliable marker of tissue blood flow^14^,^42^,^43^. Using this model, we showed that deleting mTORC1 completely blocked the elevation in blood flow normally observed in BAT chronically exposed to cold (Fig. 2L). Interestingly, we observed that the expression of vascular endothelial growth factor alpha (*Vegfa*), a key regulator of angiogenesis, was significantly reduced in BAT of Ad-Raptor^KO^ chronically exposed to cold (Figure S2G). Altogether, these results show that mTORC1 in BAT is essential to support oxidative metabolism and the normal physiological adaptation of this tissue to cold temperature.

Despite a severe decrease in BAT oxidative metabolism and systemic oxygen consumption, which supports a reduction in BAT thermogenesis, Ad-Raptor^KO^ mice survived to cold exposure. We observed signs of torpor in Ad-Raptor^KO^ mice, but this effect was marginal, being noticed in only a few mice. These results indicate that shivering may have been activated in skeletal muscle as an alternative to produce heat and preserve body temperature. Supporting this hypothesis, Ad-Raptor^KO^ mice showed higher plasma levels of creatine kinase (CK) activity, which is indicative of enhanced shivering (Fig. 2M).

### Live metabolic imaging reveals profound modifications in substrate partitioning in BAT of Ad-Raptor^KO^ mice

**Figure 3:**
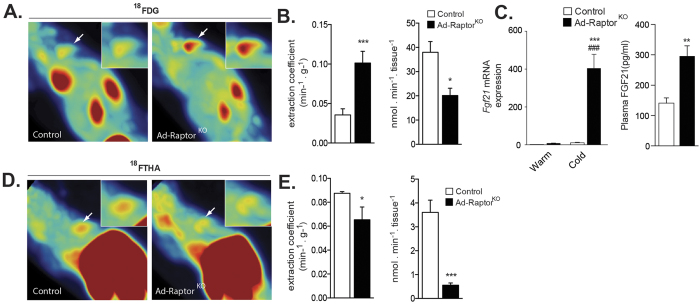
Loss of mTORC1 affects substrate partitioning in BAT. Control and Ad-Raptor^KO^ mice were exposed to cold (10 °C) during 2 weeks and fasted for 6 hours before dynamic PET scan procedures. (**A**) Representative PET images of extraction coefficient uptake of the glucose analog ^18^FDG between 25–30 min post-injection (last frame). White arrows point to the interscapular BAT that is highlighted in the upper right insert. (**B**) (Left) Glucose extraction coefficient was determined using the Patlak graphical analysis following a 30-minutes dynamic scan. (Right) Total dynamic glucose uptake corrected for tissue weight was calculated from the extraction coefficient, BAT weight and plasma glucose levels (n = 4–5; mean +/− SEM; t-test; *P < 0.05; ***P < 0.001). (**C**) Gene expression (left) (n = 4; mean +/− SEM; Two-way ANOVA; ***P < 0.01 vs. Warm; ^###^P < 0.001 vs. Control) and circulating levels (right) of FGF21 (n = 8; mean +/− SEM; t-test; **P < 0.01). (**D**) Representative PET images of the uptake of the fatty acid analog ^18^FTHA between 25–30 min post-injection (last frame). White arrows point to the interscapular BAT that is highlighted in the upper right insert. (**E**) (Left) NEFA extraction coefficient was determined using the Patlak graphical analysis following a 30-minutes dynamic scan. (Right) Total dynamic NEFA uptake corrected for tissue weight was calculated from the extraction coefficient, BAT weight and plasma NEFA levels (n = 4; mean +/− SEM; t-test; *P < 0.05; ***P < 0.001).

Glucose uptake is rapidly increased in BAT of rodents^14^,^44^,^45^ and humans^9^,^23^,^46^ exposed to cold. Glycolytic intermediates are essential to support lipogenesis and thermogenesis in BAT^47^,^48^,^49^,^50^. Here, the dynamic uptake of glucose by tissues was assessed using the PET tracer 2-deoxy-2-[^18^F]-fluoro-D-glucose (^18^FDG). Upon an acute cold challenge, we observed that ^18^FDG uptake by BAT was significantly higher in Ad-Raptor^KO^ mice versus controls (Figure S3A and S3B). In mice chronically exposed to cold, we noticed that the extraction coefficient (capacity for one gram of tissue to extract substrate from the circulation) was increased in response to mTORC1 loss (Fig. 3A and B left panel). Such increase in glucose uptake is likely the result of the elevation in Akt activation in BAT of Ad-Raptor^KO^ mice (Fig. 2A), a kinase recognized to play a crucial role in promoting glucose transport in brown adipocytes^30^,^51^. Interestingly, we also noted elevated expression of *fibroblast growth factor 21 (Fgf21*) in BAT of Ad-Raptor^KO^ mice chronically exposed to cold (Fig. 3C), which may have contributed to drive glucose uptake, as reported^52^,^53^,^54^. High circulating FGF21 was also detected in these mice (Fig. 3C). Despite the increase in glucose extraction coefficient, it is important to point out that the total uptake of glucose by the whole BAT was reduced in Ad-Raptor^KO^ (Fig. 3B, right panel). This was likely caused by the severe reduction in BAT mass and by the elevation in glucose uptake in WAT in these mice (Figure S3C).

NEFA uptake is strongly enhanced in BAT upon cold exposure^9^,^14^. The uptake of NEFA is key to support lipogenesis and the replenishment of intracellular TG pools during acute and chronic cold exposure^14^. To determine the impact of mTORC1 loss on NEFA uptake by BAT, we performed a set of experiments with the fatty-acid PET tracer analogue, [^18^F]-fluoro-6-thia-heptadecanoic acid (^18^FTHA). We first observed that ^18^FTHA uptake by BAT was not different between control and Ad-Raptor^KO^ mice acutely exposed to cold (Figure S3D,E). However, we measured a slight, but significant reduction in ^18^FTHA uptake in BAT of Ad-Raptor^KO^ mice exposed to a chronic cold challenge (Fig. 3D,E). We noticed that both the extraction coefficient and the total uptake rate were significantly decreased in BAT of Ad-Raptor^KO^ mice (Fig. 3E). These results are consistent with the fact that BAT of Ad-Raptor^KO^ mice exhibit profound reductions in mitochondrial mass, BAT expansion, and oxidative metabolism in response to chronic cold stimulation.

### Loss of mTORC1 severely impairs metabolism in BAT following chronic cold exposure

**Figure 4:**
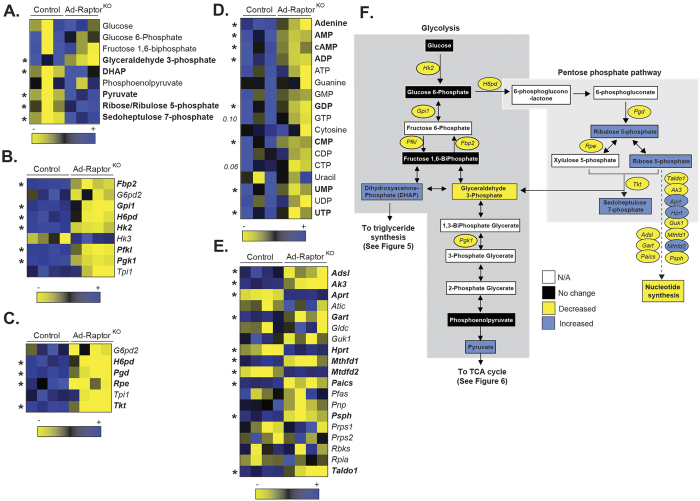
mTORC1 loss affects glucose metabolism in BAT following chronic cold exposure. Control and Ad-Raptor^KO^ mice were cold exposed during 14 days and fasted for 6 hours before BAT collection. (**A**) Glycolytic intermediates measured by metabolomics. Metabolites in bold are statistically different between control and Ad-Raptor^KO^ mice (n = 3; mean +/− SEM; t-test; *P < 0.05). (**B**) Glycolytic gene expression extracted from the microarray dataset. Genes in bold are statistically different between control and Ad-Raptor^KO^ mice (n = 4; mean +/− SEM; SAM t-test; *FDR < 0.05). (**C**) Genes expression of components of the pentose phosphate pathway extracted from the microarray dataset. Genes in bold are statistically different between control and Ad-Raptor^KO^ mice (n = 4; mean +/− SEM; SAM t-test; *FDR < 0.05). (**D**) Nucleotides measured by metabolomics. Metabolites in bold are statistically different between control and Ad-Raptor^KO^ mice (n = 3; mean +/− SEM; t-test; *P < 0.05). (**E**) Expression of genes involved in nucleotide synthesis extracted from the microarray dataset. Genes in bold are statistically different between control and Ad-Raptor^KO^ mice (n = 4; mean +/− SEM; SAM t-test; *FDR < 0.05). (**F**) Schematic summary of transcriptomics and metabolomics data.

To better define the importance of mTORC1 for BAT metabolic adaptation to cold, we have performed metabolomics and transcriptomics analyses on BAT samples isolated from control and Ad-Raptor^KO^ mice. Supporting the elevation in glucose extraction in response to chronic mTORC1 inhibition (Fig. 3), BAT of Ad-Raptor^KO^ mice markedly accumulated key glycolytic products (Fig. 4A, see also the metabolic map in Fig. 4F). Specifically, we observed a striking elevation in dihydroxyacetone-phosphate (DHAP) and pyruvate levels in Ad-Raptor^KO^ mice (3.6 and 6.4 fold, respectively). Such elevations were observed despite a reduction in the expression of many glycolytic genes, indicating that these changes were not driven by a transcriptional modulation of the glycolytic machinery (Fig. 4B). Supporting the elevation in glucose flux in response to mTORC1 loss, we also measured an increase in the production of ribose/ribulose 5-phosphate and sedoheptulose 7-phosphate, which are key intermediates of the pentose phosphate pathway (PPP) (Fig. 4A). As observed in the case of glycolysis, the elevation in PPP metabolites was associated with a reduction rather than an increase in the expression of genes coding for proteins regulating this pathway (Fig. 4C). The PPP is a key metabolic pathway used for nucleotide biosynthesis and for the generation of NADPH for *de novo* lipogenesis. Interestingly, we observed a severe decrease in the levels of most nucleotides in BAT of Ad-Raptor^KO^ mice, indicating an inability of the tissue to produce nucleotides despite the accumulation of the several molecules required for their production (Fig. 4D). At the transcriptional level, we observed a mixed profile regarding the expression of genes regulating nucleotide biosynthesis, some genes showing an elevation whereas others showing a reduction (Fig. 4E).

**Figure 5:**
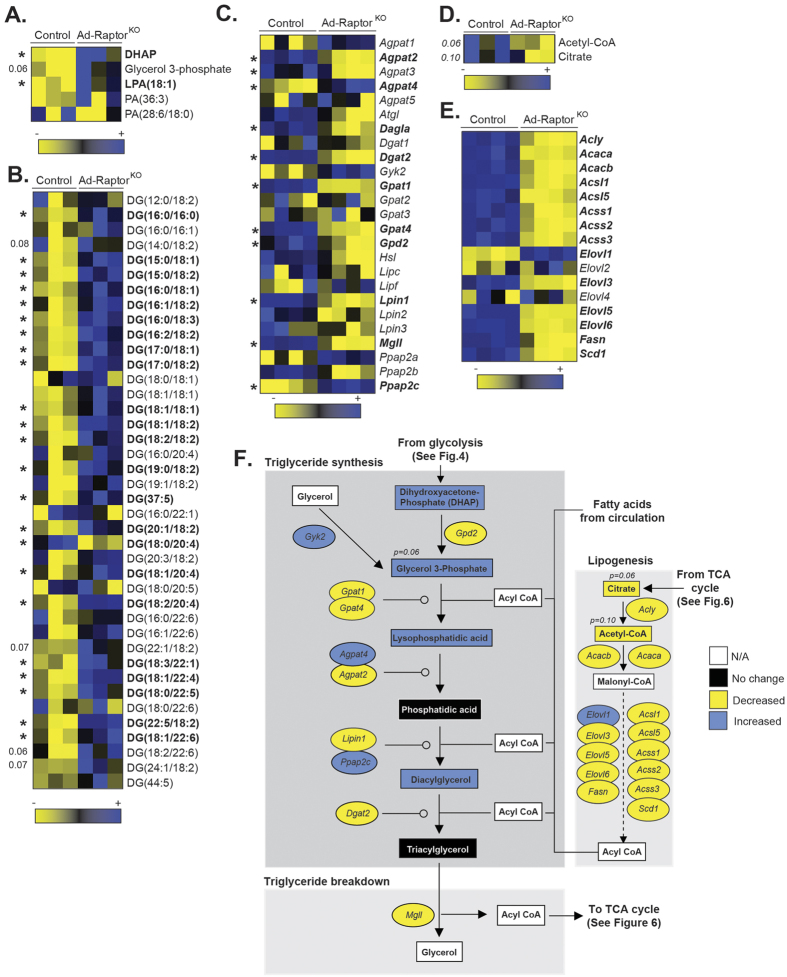
mTORC1 loss severely impairs lipid metabolism in BAT following chronic cold exposure. Control and Ad-Raptor^KO^ mice were cold exposed for 14 days and fasted during 6 hours before BAT collection. (**A**) Metabolites entering in the synthesis of glycerolipids from glucose measured by metabolomics. Metabolites in bold are statistically different between control and Ad-Raptor^KO^ mice (n = 3; mean +/− SEM; t-test; *P < 0.05). (**B**) Diacylglycerol (DG) species measured by metabolomics. Metabolites in bold are statistically different between control and Ad-Raptor^KO^ mice (n = 3; mean +/− SEM; t-test; *P < 0.05). (**C**) Expression of genes entering in the synthesis or the breakdown of glycerolipids extracted from the microarray dataset. Genes in bold are statistically different between control and Ad-Raptor^KO^ mice (n = 4; mean +/− SEM; SAM t-test; *FDR < 0.05). (**D**) Lipogenic metabolites produced from the TCA cycle measured by metabolomics (n = 3; mean +/− SEM; t-test). (**E**) Lipogenic gene expression extracted from the microarray dataset. Genes in bold are statistically different between control and Ad-Raptor^KO^ mice (n = 4; mean +/− SEM; SAM t-test; *FDR < 0.05). (**F**) Schematic summary of transcriptomics and metabolomics data.

The metabolomics data presented above showed that Ad-Raptor^KO^ mice accumulated high levels of DHAP, a key glycolytic intermediate used for the synthesis of glycerolipids. Downstream of DHAP, we observed an elevation in glycerol 3-phosphate and lysophosphatidic acid (LPA) accumulation in BAT of Ad-Raptor^KO^ mice (Fig. 5A, see also the metabolic map in Fig. 5G). This effect was linked to a dramatic increase in the content of various diacylglycerols (DG) (Fig. 5B). Although several TG species were also higher in BAT of Ad-Raptor^KO^ mice than in control animals, this trend was not as striking as what was observed for DG (Figure S4). Nevertheless, these results indicate that an important fraction of glycolytic intermediates are shuttled onto the glycerol moiety of glycerolipids in Ad-Raptor^KO^ mice. As reported above for glycolysis and PPP, these effects were not caused by an elevation in the expression of genes coding for proteins regulating this process. Indeed, we observed that several genes promoting glycerolipid synthesis were decreased rather than increased in BAT of Ad-Raptor^KO^ mice (Fig. 5C). Triacylglycerol synthesis involves the sequential esterification of acyl-CoA on glycerol 3-phosphate, phosphatidic acid, and DG respectively. The acyl-CoA molecules are provided either by circulating lipids or can be synthesized *de novo* from the conversion of citrate to malonyl-CoA (see the metabolic map in Fig. 5F). Here, we observed a reduction in citrate and acetyl-CoA levels in BAT of Ad-Raptor^KO^ (Fig. 5D). We also measured a profound defect in the expression of genes coding for proteins required for *de novo* fatty acid synthesis (Fig. 5E). These results suggest that the fatty acids contributing to the elevation in glycerolipid synthesis in BAT of Ad-Raptor^KO^ mice are most likely derived from circulating lipids rather than *de novo* lipogenesis.

**Figure 6:**
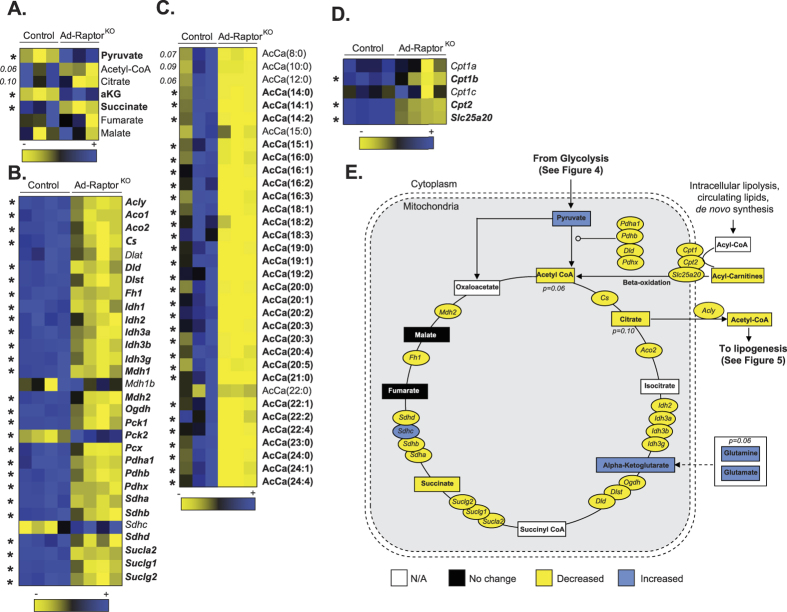
mTORC1 impairs oxidative metabolism in BAT following chronic cold exposure. Control and Ad-Raptor^KO^ mice were cold exposed for 14 days and fasted during 6 hours before BAT collection. (**A**) Metabolites of the TCA cycle measured by metabolomics. Metabolites in bold are statistically different between control and Ad-Raptor^KO^ mice (n = 3; mean +/− SEM; t-test; *P < 0.05). (**B**) TCA cycle gene expression extracted from the microarray dataset. Genes in bold are statistically different between control and Ad-Raptor^KO^ mice (n = 4; mean +/− SEM; SAM t-test; *FDR < 0.05). (**C**) Acyl-Carnitine (AcCa) metabolites measured by metabolomics. Metabolites in bold are statistically different between control and Ad-Raptor^KO^ mice (n = 3; mean +/− SEM; t-test; *P < 0.05). (**D**) Expression of genes controlling Acyl-Carnitine production extracted from the microarray dataset. Genes in bold are statistically different between control and Ad-Raptor^KO^ mice (n = 4; mean +/− SEM; SAM t-test; *FDR < 0.05). (**E**) Schematic summary of transcriptomics and metabolomics data.

As presented in Fig. 6A (and Fig. 4A), high levels of pyruvate accumulated in BAT of Ad-Raptor^KO^ mice. Pyruvate molecules produced from glycolysis can be used to produce lactate or can be transported into the mitochondria where they are converted to acetyl-CoA. In the mitochondria, acetyl-CoA is used in the TCA cycle to promote cellular respiration and support thermogenesis (see metabolic map in Fig. 6E). Despite high levels of pyruvate in BAT of Ad-Raptor^KO^ mice, we observed no change in lactate levels, but did measure a reduction in the levels of acetyl-CoA and other TCA cycle intermediates such as citrate and succinate, indicating a defect in pyruvate catabolism (Fig. 6A). This effect was associated with a profound reduction in the expression of genes coding for proteins catalyzing the conversion of pyruvate to acetyl-CoA, as well as almost all the genes encoding proteins of the TCA cycle (Fig. 6B). Interestingly, despite the clear impairment in TCA cycle, we observed that alpha-ketoglutarate (aKG) levels were elevated in Ad-Raptor^KO^ mice (Figure S5A and Fig. 6E). This effect may have been caused by the elevation in glutaminolysis, as we observed very high levels of glutamine and glutamate in Ad-Raptor^KO^ mice (Figure S5B). In addition to pyruvate, acetyl-CoA can be produced from the β-oxidation of acyl-CoA in the mitochondria. The import and catabolism of acyl-CoA depends on the production of acyl-carnitines (AcCa) by carnitine acetyltransferases. As shown in Fig. 6C, the production of AcCa was completely impaired in BAT of Ad-Raptor^KO^ mice. We also observed a significant reduction in the expression of the genes encoding proteins required for AcCa formation and mitochondrial transport (Fig. 6D). Altogether, these results indicate that glucose and fatty acid oxidative metabolism are severely reduced in BAT of Ad-Raptor^KO^ mice. An integrative representation of the metabolomics and transcriptomics data is presented in Figure S6.

### The defects in BAT expansion and metabolism in Ad-Raptor^KO^ are not mediated by a reduction in adrenergic signaling

**Figure 7:**
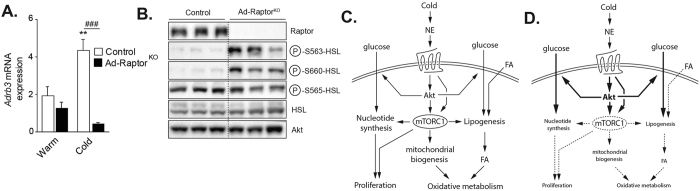
The defects in BAT expansion and metabolism in Ad-Raptor^KO^ are not mediated by a reduction in adrenergic signalling. Control and Ad-Raptor^KO^ mice were cold exposed for 14 days and fasted during 6 hours before BAT collection. (**A**) Gene expression of *Adrb3* (n = 4; mean +/− SEM; Two-way ANOVA; **P < 0.01 vs. Warm; ^###^P < 0.001 vs. Control). (**B**) Western blots performed on BAT collected from control and Ad-Raptor^KO^ mice following chronic cold exposure. All gels have been run under the same experimental conditions. (**C**) Model summarizing the role of mTORC1 in BAT during cold adaptation. (**D**) Model summarizing the impact of mTORC1 loss in BAT during cold adaptation. Bold and dashed lines indicate increase and decrease respectively, in comparison to the model presented in (**C**).

It was recently shown that chronic mTORC1 inhibition with rapamycin reduces the transcription of the β3-adrenergic receptor (*Adrb3*) in WAT and BAT^29^. To determine whether a reduction in adrenergic signaling could have contributed to the defect in BAT expansion and metabolism in Ad-Raptor^KO^ mice, we measured mRNA expression of *Adrb3.* Although mTORC1 loss did not affect *Adrb3* expression in BAT of mice kept at thermoneutrality, we observed a severe reduction in the expression of this gene when animals were chronically exposed to cold (Fig. 7A). To evaluate whether this effect was associated with changes in adrenergic signaling, we next performed western blot analyses to measure the phosphorylation status of hormone sensitive lipase (HSL) on S563 and S660, which are key residues phosphorylated downstream of ADRB3. Surprisingly, we observed that, despite a severe reduction in *Adrb3* mRNA expression, the phosphorylation of HSL on S563 and S660 was massively increased in Ad-Raptor^KO^ mice. On the other hand, the phosphorylation of HSL on S565, a site phosphorylated by AMP kinase that negatively regulates the activity of HSL, was not modulated in these conditions. These results indicate that defects in BAT observed in response to chronic mTORC1 inhibition are not a consequence of a reduction in adrenergic signaling. In fact, these changes occurred in the face of elevated adrenergic signaling, which further highlights the importance of mTORC1 for BAT recruitment and metabolic adaptation to cold. How mTORC1 inhibition drives adrenergic signaling is unknown, but does not involve any elevation in *Adrb1* or *Adrb2* expression (Figure S7). A model recapitulating our findings is presented in Fig. 7C,D.

## Discussion

In response to chronic cold exposure, BAT mass expands to increase thermogenic capacity^1^. This process, termed BAT recruitment, plays a crucial role in the long term adaptation to a cold environment. The signaling events that contribute to BAT expansion during chronic cold exposure are not completely characterized. Here we show that mTORC1 activity is highly induced in BAT following cold exposure. This effect was observed in response to acute and chronic cold stimulation, supporting a continuous role for mTORC1 in the response of BAT to cold. Over the years, nutrients and growth factors were identified as the main factors activating mTORC1^25^. The elevation in circulating levels of amino acids, glucose and insulin following food intake rapidly induces mTORC1 in tissues^39^,^55^. Here, we provide evidences that mTORC1 activation by cold occurs independently of nutrients and insulin. Hence, cold-driven mTORC1 activity was observed in mice that did not have access to food. In these animals, we did not observe any increase in insulin and glucose levels following cold exposure. Additionally, we observed that cold-induced mTORC1 activity was completely blocked when BAT was denervated. In these experiments, a direct comparison between the innervated and the denervated lobes of BAT was performed within the same mouse, thus ruling out the contribution of any humoral factor to the regulation of the pathway. Together, these results clearly demonstrate that the SNS is primarily responsible for inducing mTORC1 in mice exposed to cold.

Two recent studies reported that β-adrenergic stimulation and cold exposure activate mTORC1, an effect that was shown to be essential to support WAT browning and BAT formation^28^,^29^. Importantly, the role of mTORC1 in regulating BAT recruitment and metabolism *in vivo* in response to chronic cold stimulation, which is the object of the present report, was not addressed in these studies. One of these reports revealed that β-adrenergic stimulation activates mTORC1 by promoting the phosphorylation of Raptor by protein kinase A (PKA), an effect that was reported to be independent of Akt, a key activator of mTORC1^56^,^57^,^58^,^59^. Here, we found that the activation of mTORC1 by cold was associated with an increase in Akt phosphorylation. The reason for these differences is unclear but suggests that the SNS likely activates mTORC1 through Akt-dependent and Akt-independent processes. The phosphorylation of Akt on S473 is an event that is mediated by mTORC2^60^. Consistent with our findings, a recent report shows that the mTORC2/Akt axis is activated in brown adipocytes upon β-adrenergic stimulation and cold exposure^30^. In this study, adipose-specific loss of mTORC2 was shown to reduce Akt-mediated glucose uptake and to lower resistance to cold^30^. Another report also showed that adrenergic stimulation promotes mTORC2 activity in brown adipocytes *in vitro*^61^. In that case, it was observed that mTORC2 promotes glucose uptake independently of Akt^61^. Altogether, these evidence indicate that cold sequentially activates mTORC2 and mTORC1, and suggest that each of these complexes plays complementary roles to insure the optimal tissue response to cold. On the one hand, mTORC2 promotes glucose uptake, an event required to support lipogenesis and thermogenesis^14^,^61^. On the other hand, mTORC1 promotes BAT expansion, mitochondrial biogenesis and oxidative metabolism, which are all needed to sustain heat production. It is extremely important to take into consideration that cold exposure rapidly drives food intake in rodents to cope with the rise in energy expenditure^1^. Since the mTOR pathway is very sensitive to activation by insulin and nutrients, it is likely that nutrition synergizes with the adrenergic stimulation to maximize the activation of mTOR signaling in response to cold.

Our findings clearly show that mTORC1 deeply affects mitochondrial mass. We report that cold-induced mitochondrial biogenesis is completely impaired in Ad-Raptor^KO^ mice, an effect associated with an important reduction in the expression of *Pgc1a*, a transcriptional co-activator that controls mitochondrial biogenesis and function^62^. Interestingly, mice with heart- or skeletal muscle-specific deletion of Raptor also exhibit a reduction in mitochondrial mass and *Pgc1a* expression^63^,^64^,^65^. Consistent with the reduction in mitochondrial number, live metabolic imaging and indirect calorimetry experiments revealed a severe defect in BAT oxidative metabolism and systemic oxygen consumption in Ad-Raptor^KO^ mice. Of note, we showed that the rise in oxygen consumption observed in response to acute cold exposure was significantly reduced in Ad-Raptor^KO^ mice. Strikingly, the additional elevation in oxygen consumption in mice chronically exposed to cold, a phenomenon linked to the increase in BAT mass and thermogenic capacity, was completely prevented in mice lacking mTORC1. These results demonstrate that mTORC1 is required to support mitochondrial biogenesis and to increase the thermogenic potential of BAT in response to chronic cold exposure. It is interesting to note that the reduction in mitochondrial biogenesis and the impairment in oxidative metabolism observed in Ad-Raptor^KO^ mice were associated with reductions in *Vegfa* expression and blood flow in BAT. The importance of mTORC1 in regulating angiogenesis has been demonstrated over the years, especially in the context of tumorigenesis^66^. Here, our results indicate that mTORC1 exerts similar pro-angiogenic function in BAT *in vivo*.

Despite a clear defect in BAT oxidative metabolism, all Ad-Raptor^KO^ survived exposure to cold. We noted that a few Ad-Raptor^KO^ mice showed signs of torpor in response to cold exposure but no animal died, indicating that alternative heat-producing processes were taking place to ensure survival. Accordingly, Ad-Raptor^KO^ mice showed high plasma levels of CK activity, a surrogate marker of shivering^29^,^67^. Other mouse models with defective BAT thermogenesis have also been shown to survive in the cold by promoting shivering^68^,^69^.

Using live metabolic imaging, we showed that glucose uptake was increased in BAT of Ad-Raptor^KO^ mice. The elevation in Akt phosphorylation, an effect linked to the reduction in the negative feedback loops from mTORC1 to PI3K-Akt, likely contributed to stimulate glucose uptake. We also measured a spectacular induction in *Fgf21* expression in BAT of Ad-Raptor^KO^ mice exposed to cold. This growth factor, that was identified as a key regulator of glucose uptake and metabolism^52^,^53^,^54^, was previously shown to be produced and secreted by various tissues, including BAT^70^,^71^. Interestingly, a very similar induction in *Fgf21* expression was observed in BAT of Ucp1^KO^ mice^71^, suggesting that elevated *Fgf21* production may be a common response to impaired BAT thermogenesis. Supporting a rise in glucose flux, we observed that the levels of the glycolytic intermediate DHAP were higher in BAT of Ad-Raptor^KO^ mice. The elevation in DHAP was concomitant to an increase in glycerol 3-phosphate, LPA and DG, indicating that a significant fraction of glycolytic intermediates was channelled to the glycerol moiety of glycerolipids. Importantly, the increase in lipid synthesis was probably not the only factor that has exacerbated lipid deposition in BAT of Ad-Raptor^KO^ mice. The reduction in oxidative metabolism, a direct consequence of impaired mitochondrial biogenesis and TCA cycle activity, has likely contributed to reduce the degradation of lipids in BAT of Ad-Raptor^KO^ mice. In addition to DHAP, we also observed that pyruvate, another glycolytic intermediate, accumulated in BAT of Ad-Raptor^KO^ mice. As observed for DHAP, the rise in pyruvate levels was likely the result of both increased glycolytic flux and reduced pyruvate oxidation. We observed that the conversion of pyruvate to acetyl-CoA, a step required for the oxidation of pyruvate in the mitochondria, was severely blocked in BAT of Ad-Raptor^KO^ mice. Altogether, these findings highlight the severity of the mitochondrial defects induced by chronic mTORC1 deletion.

It was recently shown that cold increases the expression of genes related to PPP in BAT^72^, a metabolic pathway required to sustain *de novo* lipogenesis and nucleotide biosynthesis. Here, we confirmed these findings in control mice. Interestingly, Ad-Raptor^KO^ mice showed a significant reduction in the expression of PPP genes. Despite such defects, the amount of PPP intermediates was higher in BAT of these mice. The reason for this apparent discrepancy is unclear, but may be a consequence of elevated glucose flux in BAT of Ad-Raptor^KO^ mice. Interestingly, despite elevated PPP intermediates, we observed a striking reduction in the production of nucleotides in BAT of Ad-Raptor^KO^ mice, suggesting a defect in nucleotide biosynthetic capability in the absence of mTORC1. Interestingly, recent reports indicate that mTORC1 directly regulates nucleotide synthesis^73^,^74^. These studies show that mTORC1 stimulates the production of new nucleotides to accommodate an increase in RNA and DNA synthesis needed for ribosome biogenesis and anabolic growth. Here, we provide evidence that mTORC1 plays a crucial role in controlling the biosynthesis of nucleotides in BAT. In addition to its well-established roles in promoting protein and lipid synthesis^25^, which are both required to support cell growth and proliferation, mTORC1 could regulate BAT expansion also by promoting nucleotide synthesis.

Mice lacking mTORC1 in adipose tissue were previously generated by another group by crossing *Raptor* floxed mice with Ap2-Cre mice (Ap2-Raptor^KO^)^38^. Although the implication of mTORC1 in regulating BAT expansion and metabolism in response to cold was not tested in this report, a few observations were made regarding the role of mTORC1 on systemic metabolism and BAT function. These investigators reported that BAT weight is reduced in Ap2-Cre mice, similar to what is reported here. Nevertheless, no obvious morphological change was reported in BAT of the Ap2-Cre model. Despite a reduction in BAT mass, the Ap2-Raptor^KO^ mice showed enhanced oxygen consumption and protection against high-fat diet induced obesity when housed at room temperature^38^. Although BAT oxidative metabolism and thermogenic potential were not directly assessed in these mice, these results appear to oppose our findings. The reason for these differences is unclear but could likely be related to the use of the Ap2-Cre model, which was shown to mediate the recombination of floxed alleles in non-adipocyte cell types^75^,^76^. Another group recently developed mice lacking mTORC1 in adipose tissue using the adiponectin-cre mouse^77^, exactly as described here. In this study, Ad-Raptor^KO^ housed at room temperature showed a reduction in WAT mass. As reported by Polak *et al.*, the authors found that Ad-Raptor^KO^ mice are protected against high-fat diet-induced obesity. However, they found that this effect was not associated with change in energy expenditure. This last results is in accordance with our experiments showing that oxygen consumption is minimally affected when Ad-Raptor^KO^ are not exposed to cold. Although Lee *et al.* did not test the impact of cold on BAT in Ad-Raptor^KO^ mice, they report that mTORC1 loss reduces BAT mass, an effect associated with elevated lipid deposition in brown adipocytes^77^. Moreover, they observed that deleting Raptor only in brown adipocytes using the UCP1-cre mouse similarly affected BAT mass, indicating that mTORC1 cell-autonomously controls the development of this tissue.

Recent studies reported the impact of *tuberous sclerosis complex 1 (Tsc1*) loss on WAT and BAT^78^,^79^. Because the loss of TSC1 constitutively activates mTORC1, it would be expected that these studies would show results that oppose our findings. Accordingly, Adiponectin-cre mediated recombination of *Tsc1* increased BAT mass, an effect associated with a trend towards higher BAT DNA content^78^. The impact of cold on BAT mass expansion and metabolism was not tested in this report. In another study, Xiang *et al.* depleted *Tsc1* in adipose tissue using the Ap2-cre mice^79^. In this report, constitutive mTORC1 activation led to a reduction in *Ucp1* and *Pgc1a* expression, a decrease in mitochondrial DNA content and an increase in lipid deposition in BAT. Although these results oppose our findings, it is important to point out that Ap2-Tsc1^KO^ mice die from severe lungs dysfunction 48 hours after birth^80^. Investigating BAT function and morphology in these conditions was probably not optimal and preclude any comparison with our study.

Altogether, our results indicate that mTORC1 loss impairs BAT recruitment in response to chronic cold exposure, an effect associated with profound defects in mitochondrial biogenesis and metabolism. Although these results clearly establish mTORC1 as an important regulator of BAT function, it is important to point out that these observations were made following the chronic inhibition of mTORC1 in the developing adipocyte, and that we cannot exclude the possibility that some effects reported were secondary to the long-term impairment of mTORC1 signaling. Defining the impact of acute Raptor deletion using an inducible system to deplete mTORC1 only in BAT of adult mice would represent a valuable complement to the present study.

In conclusion, we provide the evidence that mTORC1 plays a critical role in the control of BAT recruitment and metabolism in response to cold. The present results extend the understanding of the molecular mechanisms regulating thermogenesis and provide possible avenues for the development of tools to control energy expenditure, fight obesity and improve glucose and lipid homeostasis in humans.

## Experimental Procedures

### Animal care

Male C57Bl/6N (10–12 weeks old) mice weighing 20–25 g (Jackson Laboratory, ME, USA) were divided into two groups, which were (i) kept at thermoneutrality (30 °C) or (ii) housed at 10 °C, for 14 days. Raptor floxed mice were developed previously and ordered from Jackson Laboratory (stock number 013188). Adiponectin-cre mice were also ordered from Jackson Laboratory (stock number 010803)^39^. For all experiments, male *adiponectin*-Cre; *raptor*^LoxP/LoxP^ (Ad-Raptor^KO^) and *raptor*^LoxP/LoxP^ (control) mice aged between 8 and 14 weeks were used. All animals were kept under a 12 h light/dark cycle (lights on at 0600 hours) with *ad libitum* access to pelleted chow (Rodent Laboratory Chow 5001, Purina, St. Louis, MO) and tap water. Distinct groups of animals were used for the PET procedures and immunohistochemistry/biochemical measurements described below. All experimental protocols were approved by the Animal Ethics Committee of the *Université de Sherbrooke* and in accordance with the guidelines of the Canadian Council on Animal Care.

### Cold exposition

Following warm (30 °C) or cold exposure (10 °C), mice were individually housed in new cages without access to food but with free access to water. Mice were fasted during 6 h. In some experiments, mice were injected intraperitoneally with rapamycin (2 mg/kg, Sigma #37094) 1 h prior to the 6 h cold exposure.

### Unilateral BAT denervation

Unilateral BAT denervation was performed as previously described^81^,^82^. Eight weeks old mice were used for this experiment. On the day of surgery, each mouse was weighed and anesthetized with isoflurane. The mouse was shaved and secured on a warm surgical table. Following a standard skin disinfection procedure with ethanol and chlorhexidine (Baxter), a lateral incision was made to expose the interscapular fat pads. A blunt forceps was then used to clamp the caudal edge of the interscapular fat pad and then retract cranially over the head of the mouse. All five branches of intercostal sympathetic nerves connecting to the right BAT fat pad were identified, carefully isolated, and sectioned. After BAT denervation, the interscapular fat pad was returned to its original position. Mice were monitored for 7 days post surgery before being exposed to experimental conditions.

### Blood and tissue sampling

Mice were fasted for 6 h (06:00–12:00) and were euthanized through cardiac puncture under isoflurane anaesthesia and samples were transferred into EDTA-coated tubes. Plasma glucose, NEFA and TG levels were measured as described previously^14^. Insulin, leptin and FGF21 were measured using ELISA kits (#90080 and #90030, Crystal Chem, Downers Grove, IL; #MF2001, R&D Systems, Minneapolis, NE). Creatine kinase activity was measured using the Creatine Kinase Activity Assay Kit (#MAK116, Sigma Aldrich, MO). BAT was carefully excised, dissected on ice and weighed. BAT samples were immediately frozen in liquid nitrogen for protein, RNA and DNA extractions or fixed in 4% (wt/vol) paraformaldehyde for histological analyses.

### Western blotting

Tissues were lysed with Triton-X 100 containing lysis buffer (50 mM HEPES, pH 7.4, 2 mM EDTA, 10 mM sodium pyrophosphate, 10 mM sodium glycerophosphate, 40 mM NaCl, 50 mM NaF, 2 mM sodium orthovanadate, 1% Triton-X 100, 0.1% sodium lauryl sulfate, 1% sodium deoxycholate and one tablet of EDTA-free protease inhibitors Roche per 25 ml). Samples were rotated at 4 °C for 10 minutes and then the soluble fractions of cell lysates were isolated by centrifugation at maximum speed for 10 min in a microcentrifuge. Protein levels were then quantified using Bradford reagents and analyzed by Western blotting. Samples were loaded on 4–12% Nupage precast gels or 10% Tris-Glycine gels (Life Technologies). Proteins were transferred to PVDF membranes and incubated with the primary antibody overnight at 4 °C in 5% non-fat milk diluted in PBS-Tween. The following antibodies were used: Akt (Cell Signaling Technology, 4691, dilution 1:1000), phospho-AKT T308 (Cell Signaling Technology, 2965, dilution 1:1000), phospho-AKT S473 (Cell Signaling Technology, 9271, dilution 1:1000), S6 (Cell Signaling Technology, 2217, dilution 1:2500), phospho-S6 S240/244 (Cell Signaling Technology, 5364, dilution 1:1000), Raptor (Cell Signaling Technology, 2280, dilution 1:1000), HSL (Cell Signaling Technology, 4107, dilution 1:1000), phospho-HSL S563 (Cell Signaling Technology, 4139, dilution 1:1000), phospho-HSL S660 (Cell Signaling Technology, 4126, dilution 1:1000), phospho-HSL S565 (Cell Signaling Technology, 4137, dilution 1:1000), UCP1 (Abcam, 10983, dilution 1:1000), and mitochondrial complexes I to V (Abcam, 110413, dilution 1:250). Secondary antibodies were purchased from Cell Signaling Technology and diluted 1:5000. Amersham ECL Western Blotting Detection Reagent (RPN2106) was used to image the blots.

### Gene expression analysis

Total RNA was isolated from BAT using PureL and the Aurum™ Total RNA Fatty and Fibrous Tissue Kit (Bio-Rad, 732–6830, Mississauga, ON, Canada). RNA concentration was estimated from absorbance at 260 nm. cDNA synthesis was performed using the iScript Advanced cDNA Synthesis Kit (Bio-Rad, 172–5038, Mississauga, ON, Canada). mRNA extraction and cDNA synthesis were performed following the manufacturer’s instructions. Relative quantification of gene expression was performed on diluted cDNA (1:20) in duplicate samples using a CFX384 touchTM real-time PCR (Bio-Rad, Mississauga, ON, Canada). Chemical detection of the PCR products was achieved with SYBR Green (Bio-Rad, 172–5271, Mississauga, ON, Canada). Specificity of amplification was ensured using melt curve analyses followed by agarose gel electrophoresis of the amplified products. Fold differences in targeted mRNA expression were calculated using 2-delta cycle threshold method and data were normalized on beta-microglobulin (*B2m*) expression.

### Measurement of Mitochondrial DNA Content

Tissues for assessing mitochondrial DNA (mtDNA) content were prepared as described previously^83^,^84^. Briefly, total DNA was isolated by proteinase K digestion followed by phenol/chloroform extraction and ethanol precipitation. Ten ng of total DNA was used to amplify mtDNA-encoded NADH dehydrogenase I (ND1) and nuclear DNA-encoded Beta-actin using real-time PCR. MtDNA content was calculated from the ratio of ND1 to genomic Beta-actin.

### Histology

BAT samples were fixed overnight in 4% paraformaldehyde in PBS at 4 °C, dehydrated, embedded in paraffin, and cut into 10 μm-thick sections. Sections were stained with hematoxylin and eosin (H&E) to perform general histology. All pictures were taken on an Olympus BX60 microscope (Tokyo, Japan) at a magnification of 40X.

### Indirect calorimetry

The Promethion High-Definition Room Calorimetry System was used for the indirect calorimetry studies (GA3, Sable Systems. Las Vegas, NV). Data acquisition and instrument control were coordinated by MetaScreen v. 1.6.2 and the obtained raw data was processed using ExpeData v. 1.4.3 (Sable Systems, Las Vegas, NV) using an analysis script detailing all aspects of data transformation. A standard 12 h light/dark cycle (6:00–12:00) was maintained throughout the calorimetry studies. Prior to data collection, all animals were acclimated to cages for 7 days. Mice were subsequently placed in metabolic cages at thermoneutrality. Mice were prior subjected to a 48 h measurement at 30 °C following a switch at 10 °C for 24 h. At the end of the procedure, mice were returned to a 10 °C environment for 10 days before a second round of measurement at 10 °C.

### Small animal PET-CT protocol

All *in vivo* PET/CT experiments were initiated immediately after the insertion of a cannula in the tail vein for injection of PET tracers. All imaging experiments were performed on the avalanche photodiode-based small animal PET scanner (LabPET/Triumph, Gamma Medica, Northridge, CA) of the Sherbrooke Molecular Imaging Centre, having a 7.5 cm axial field-of-view. The animals were anaesthetized with isoflurane (1.75%, 1.5 L/min) delivered through a nose cone, and were placed in the prone position on the scanner bed with the heart centered within the field-of-view of the scanner to include the interscapular region. Boluses of each radiopharmaceutical compound (10 MBq, in 0.2 ml of 0.9% NaCl) were injected via the caudal vein over 30 seconds after starting PET data acquisition (^11^C-acetate) and over 60 seconds for ^18^FDG and ^18^FTHA. In one set of experiments, a 20-min dynamic data acquisition with ^11^C-acetate was done to determine tissue blood flow and oxidative metabolism. Just prior to the injection of ^11^C-acetate (less than 1 minute), an intravenous injection of CL (2 mg/kg, Sigma, #C5976) was performed to measure maximal oxidative activity.

In a second set of experiments, a 30-min dynamic data acquisition with either ^18^FDG or ^18^FTHA were used to also determine glucose or NEFA utilization, respectively, as previously described^14^. List-mode dynamic data acquisition allowed for flexible time framing of the data for kinetic modeling of all tracers. Low dose CT scan imaging was performed using the integrated X-O small-animal CT scanner of the Triumph platform, consisting of a 40 W X-ray tube with a 75 μm focal spot diameter and a 2240 × 2368 CsI flat panel X-ray detector. The detector pixel size was 50 μm, and a 2 × 2 pixel binning scheme was used. Scans were performed at 60 kVp and 230 μA using 512 projections in fly mode to reduce exposure. Blood samples were taken at the end of experiments by heart punctures.

### PET imaging data analysis

For ^11^C-acetate images, dynamic series of 28 frames (1 × 30 s, 12 × 10 s, 8 × 30 s, 6 × 90 s, and 1 × 300 s) were sorted out, whereas 30 frames (1 × 30 s, 12 × 10 s, 8 × 30 s, 6 × 90 s, and 3 × 300 s) were used for ^18^FDG and ^18^FTHA imaging. 3D images were reconstructed using 15 iterations of a maximum-likelihood expectation-maximization (MLEM) algorithm incorporating physical description of the detector response functions. Regions of interest (ROIs) were drawn on short-axis images and confirmed with the μCT scan^14^. Input curves were extracted by means of a ROI drawn on the left ventricular cavity blood pool in summed last-frame images to seek better contrast. The sizes of these almost-circular ROIs were compared with images of eight cylinders of different diameters from which a recovery factor was extracted and applied to the ROIs for partial volume correction^85^. For ^11^C-acetate, we used a three-compartment kinetic model that estimates the generation of CO_2_ from the citric acid cycle using the *k*_2_ constant^42^ and the tissue blood flow through the *K*_1_ constant^42^,^43^. Glucose and NEFA extraction coefficient were determined using the Patlak graphical analysis^86^. These values represent the proportion of circulating substrate taken up per mass of tissue. The dynamic glucose and NEFA uptake were determined using the extraction coefficient multiplied by the amount of substrate in circulation.

### Metabolomics and lipidomics

Polar and lipid metabolites were extracted using a chloroform-methanol technique. Briefly, 10–30 mg of BAT were crushed to powder in a liquid nitrogen-chilled mortar and pestle. Six hundred μl of LC/MS grade methanol (containing phenylalanine-d8 and valine-d8 as internal standards) was added to BAT powder following by 300 μl LC/MS grade water and 400 μl HPLC grade chloroform (containing internal standards). Top layer containing the polar metabolites and bottom layer containing the organic/lipid metabolites were separated and dried using a speedvac. Once dried, both fractions were stored at −80 °C for further analysis. Polar metabolites were resuspended in 100 μl LC/MS grade water, vortexed for 10 min at 4 °C, centrifuged at 15,000× g in a microcentrifuge at 4 °C, and the supernatant was transferred to an LC/MS vial. 1 μl of each sample was injected for LC/MS analysis as described^87^. Metabolites were identified by matching exact mass and chromatographic retention time to an in-house library of authentic chemical standards. Data were analyzed using XCalibur QuanBrowser 2.2; raw LC/MS peak areas were normalized to the appropriate internal standard to calculate peak area ratios. A pooled biological sample was generated for quality control purposes and was injected in triplicate at 1 μl, in singlicate at 3 μl, and in singlicate at 1 μl of a 0.1X and a 0.3X dilution in water. The pre-determined quality control cutoffs for each metabolite were CV<0.25 for the peak area ratio across the triplicate injections, and R > 0.975 for the raw peak area across the dilution series (including the average of the triplicate injections). The peak area ratios were further normalized to tissue sample weight.

Organic/lipid extracts were separated on an Ascentis Express C18 2.1 × 150 mm 2.7 μm column (Sigma-Aldrich, St. Louis, MO) connected to a Dionex UltiMate 3000 UPLC system and a QExactive benchtop orbitrap mass spectrometer (Thermo Fisher Scientific, San Jose, CA) equipped with a heated electrospray ionization (HESI) probe. Dried lipid samples were typically dissolved in 50 μl acetonitrile:isopropanol:water (65:30:5) and 5 μl was injected into the LC/MS, with separate injections for positive and negative ionization modes. Mobile phase A in the chromatographic method consisted of water:ACN (60:40) in 10 mM ammonium formate and 0.1% formic acid, and mobile phase B consisted of isopropanol:acetonitrile (90:10), also with 10 mM ammonium formate and 0.1% formic acid. The chromatographic gradient was described previously^88^. The column oven and autosampler tray were held at 55 °C and 4 °C, respectively. The MS instrument parameters were as described previously^89^. The spray voltage was set to 4.2 kV, and the heated capillary and the HESI were held at 320 °C and 300 °C, respectively. The S-lens RF level was set to 50, and the sheath and auxiliary gas were set to 35 and 3 units, respectively. These conditions were held constant for both positive and negative ionization mode acquisitions. External mass calibration was performed using the standard calibration mixture every 7 days.

MS spectra of lipids were acquired in full-scan/data-dependent MS^2^ mode as previously described^90^.

### Lipidomic data analysis

High-throughput identification and relative quantification of lipids was performed separately for positive and negative ionization mode data using LipidSearch software (Thermo Fisher Scientific/Mitsui Knowledge Industries)^91^,^92^ using the default parameters for QExactive. Product Search and Alignment. After alignment, raw peak areas for all identified lipids were exported to Excel and filtered according to the following pre-determined quality control criteria: Rej (“Reject” parameter calculated by LipidSearch software) equal to 0; PQ (“Peak Quality” parameter calculated by LipidSearch software) greater than 0.85; CV (standard deviation/average peak area across triplicate injections of a representative [pooled] biological sample) below 0.4; R (linear correlation across a three-point dilution series of the representative [pooled] biological sample) greater than 0.9. Typically ~70% of identified lipids passed all four quality control criteria. Raw peak areas of the filtered lipids were added to generate a “total lipid signal” for each sample, and individual lipid peak areas were normalized to this total signal as a control for extraction efficiency and sample loading.

### Microarray analyses

Gene expression was evaluated in BAT of two groups of four mice. Whole-genome gene expression was performed using the Affymetrix GeneChip Mouse Gene 2.0 ST Array. The RNA was labeled and hybridized using a standard Affymetrix protocol performed at the Centre for Applied Genomics, Hospital for Sick Children, Toronto, Canada. The quality of arrays was judged using standard quality control parameters^93^,^94^ and all arrays passed the quality control filters. Expression values were extracted using the Robust Multichip Average (RMA) method^95^ implemented in the oligo package in R^96^. The Significance Analysis of Microarrays (SAM) method^97^ was used to identify probesets differentially expressed between groups. The false discovery rate (FDR) and the fold change threshold were set at 5% and 1.5, respectively. All analyses were carried out with the R statistical software version 3.2.3 and Bioconductor packages^98^.

### Statistical analyses

Results are expressed as means ± SEM. Comparisons were done on normally distributed data using Student’s t-test or two-way ANOVA followed by Bonferroni post hoc tests to assess the differences between the various treatments with Graph Pad Prism Software version 6.0 h for Mac (San Diego, CA, USA). Differences of *P* < 0.05 were considered statistically significant.

## Additional Information

**How to cite this article**: Labbé, S. M. *et al.* mTORC1 is Required for Brown Adipose Tissue Recruitment and Metabolic Adaptation to Cold. *Sci. Rep.* **6**, 37223; doi: 10.1038/srep37223 (2016).

**Publisher’s note:** Springer Nature remains neutral with regard to jurisdictional claims in published maps and institutional affiliations.

## Supplementary Material

Supplementary Information
