# Supplementary material for: mTORC1 is Required for Brown Adipose Tissue Recruitment and Metabolic Adaptation to Cold
Source: Sci Rep. 2016 Nov 23;6:37223. doi: 10.1038/srep37223 (PMC5120333; doi:10.1038/srep37223)
Supplement: Supplementary Information [file srep37223-s1.pdf]

SUPPLEMENTARY INFORMATIONS

mTORC1 IS REQUIRED FOR BROWN ADIPOSE TISSUE RECRUITMENT AND METABOLIC ADAPTATION TO COLD

Sébastien M. Labbé[1,2], Mathilde Mouchiroud[1,2], Alexandre Caron[1,2], Blandine Secco[1,2], Elizaveta Freinkman[3], Guillaume Lamoureux[1,2], Yves Gélinas[1,2], Roger Lecomte[4], Yohan Bossé[1], Patricia Chimin[5], William T. Festuccia[5], Denis Richard[1,2], Mathieu Laplante[1,2*]

Figure S1

Mice were either kept at thermoneutrality (warm; 30°C) or cold exposed (10°C) for 2 weeks. Acute cold (6-hour at 10°C) exposure was also performed following the thermoneutrality. (A) Angiogenic, oxidative, and lipogenic genes expression in BAT (n=8 for warm; n=5 for acute cold; n=8 for chronic cold; mean +/- SEM; ANOVA; * $P < 0.05$; ** $P < 0.01$; *** $P < 0.001$). (B) Cumulative food intake during acute cold exposure (n=7-8; mean +/- SEM; t-test; * $P< 0.05$; ** $P < 0.01$; *** $P < 0.001$). (C) Plasma metabolites following a 6-hour fasting period (n=8-16; mean +/- SEM; t-test; * $P < 0.05$; ** $P < 0.01$; *** $P < 0.001$). (D) Western blots performed on BAT collected from mice injected with CL316,243 (CL; 2 mg/kg). Mice were fasted during 6 hours before the CL injection (BAT was collected 1 hour following the injection). All gels have been run under the same experimental conditions. (E) Plasma insulin levels 1 hour following the CL injection. (F) Representative PET image showing ^{18}FDG accumulation in innervated and denervated BAT lobe following chronic cold exposure. (G) Representative H&E staining of innervated and denervated BAT lobe following chronic cold exposure. (H) mtDNA content presented as fold change versus sham-operated mice kept at thermoneutrality (n=4-10; mean +/- SEM; Two-way ANOVA; *** $P < 0.001$ vs. Warm; ### $P < 0.001$ vs. Control).

Figure S2

Control and Ad-Raptor^{KO} mice were either kept at thermoneutrality (warm; 30°C) or cold exposed (10°C) for 2 weeks. Acute cold (6 hours at 10°C) exposure was also performed following the thermoneutrality. (A) Western blots for RAPTOR protein expression in different tissues collected from control or Ad-Raptor^{KO} mice. All gels have been run under the same experimental conditions. (B) (Left) Body weight, (middle) epididimal WAT weight and (right) inguinal WAT weight of control and Ad-Raptor^{KO} mice. (C) Plasma metabolites following a 6-hour fasting period (n=8-16; mean +/- SEM; Two-way ANOVA; * P < 0.05; ** P < 0.01; *** P < 0.001 vs. Control-Warm; # P < 0.05; ### P < 0.001 vs. Control; & P < 0.05; && P < 0.01; &&& P < 0.001 vs. KO-Warm). (D) *Ucp1* gene expression in BAT (n=8; mean +/- SEM; Two-way ANOVA; *** P < 0.001 vs. Warm; ### P < 0.001 vs. Control). (E) Mean SUV time-activity curves of ^{11}C-acetate in iWAT following a dynamic PET acquisition during (left) acute and (right) chronic cold exposure (n=4-5; mean +/- SEM). Mice received an acute CL injection (2 mg/kg intravenously) 1 minute prior to the PET procedure. (F) Mean SUV time-activity curves of 11C-acetate in eWAT following a dynamic PET acquisition during (left) acute and (right) chronic cold exposure (n=4-5; mean +/- SEM). Mice received an acute CL injection (2 mg/kg intravenously) 1 minute prior to the PET procedure. (G) *Vegfa* gene expression in BAT of mice chronically exposed to cold (n=4; mean +/- SEM; t-test; * P < 0.05 vs. Control).

Figure S3

Control and Ad-Raptor^{KO} mice were kept at thermoneutrality (warm; 30°C) for 2 weeks. Following the adaptation, mice were fasted and cold-exposed for 6 hours (A, B and D, E) or for 14 days (C) before dynamic PET scan procedures or tissue analyses. (A) Representative PET images of extraction coefficient uptake of the glucose analog 18FDG between 25-30 min post-injection (last frame). White arrows point to the interscapular BAT. The white square in the right corner specifically highlights this region. (B) (Left) Glucose extraction coefficient was determined using Patlak graphical analysis following a 30-minutes dynamic scan. (Right) Total dynamic glucose uptake corrected for tissue weight was calculated from the extraction coefficient, BAT weight and plasma glucose levels (n=3; mean +/- SEM; t-test; *** P < 0.001). (C) Inguinal WAT (iWAT) total dynamic glucose uptake (left) and epididymal WAT (eWAT) total dynamic glucose uptake (right) corrected for tissue weight was calculated from the extraction coefficient, iWAT and eWAT weight and plasma glucose levels (n=4-5; mean +/- SEM; t-test; * P < 0.05). (D) Representative PET images of extraction coefficient uptake of the fatty acid analog ^{18}FTHA between 25-30 minutes post-injection (last frame). White arrows point to BAT. The white square in the right corner specifically highlights this region. (E) (Left) NEFA extraction coefficient was determined using Patlak graphical analysis following a 30-minutes dynamic scan. (Right) Total dynamic NEFA uptake corrected for tissue weight was calculated from the extraction coefficient, BAT weight and plasma NEFA levels (n=4-5; mean +/- SEM; t-test).

Figure S4

Control and Ad-Raptor^{KO} mice were cold exposed for 14 days and fasted during 6 hours before BAT collection. (A) Triacylglycerol (TG) species measured by metabolomics that were significantly different between control and Ad-RaptorKO mice. Metabolites in bold are statistically different between control and Ad-RaptorKO mice (n=3; t-test; * P < 0.05). (B) Triacylglycerol (TG) species measured by metabolomics that were not significantly different between control and Ad-RaptorKO mice. (n=3; t-test).

Figure S5

Control and Ad-Raptor^{KO} mice were cold exposed for 14 days and fasted during 6 hours before BAT collection. (A) alpha-Ketoglutarate levels measured by metabolomics (n=3; mean +/- SEM; t-test; * P < 0.05). (B) Glutamate and glutamine levels measured by metabolomics (n=3; mean +/- SEM; t-test; * P < 0.05).

Figure S6

Integrative transcriptomics and metabolomics metabolic pathways following chronic cold exposure.

Figure S7

Control and Ad-Raptor^{KO} mice were cold exposed for 14 days and fasted during 6 hours before BAT collection. Gene expression of (A) *Adrb1* and (B) *Adrb2* (n=4; mean +/- SEM; Two-way ANOVA; * P < 0.05 vs. Warm; ### P < 0.001 vs. Control).

Figure S1

A

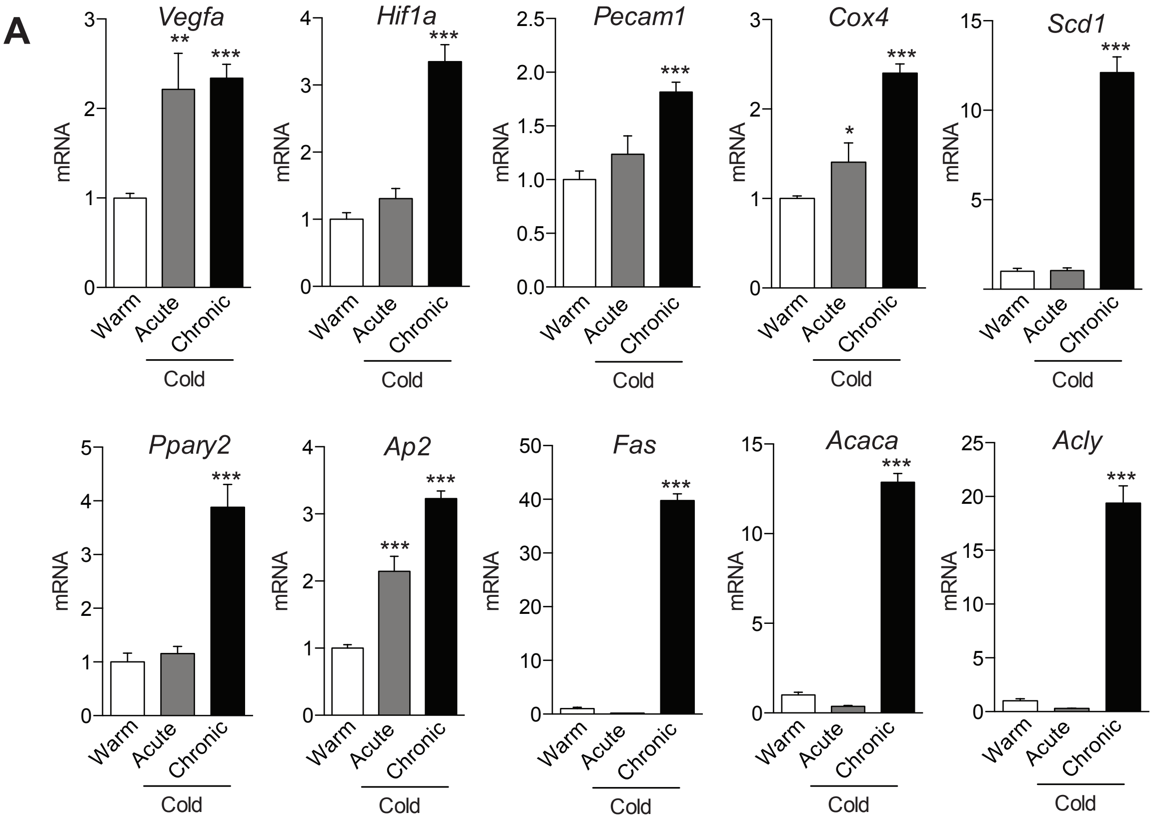

B

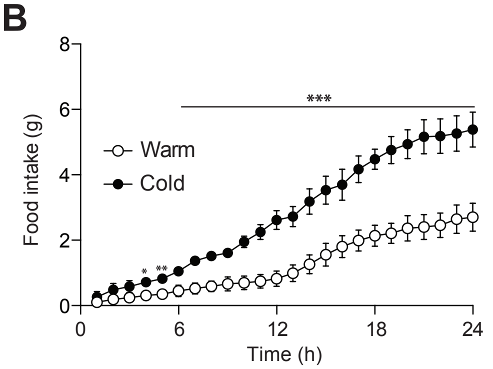

C

	control	cold	
		acute	chronic
Glucose (mmol/L)	8.8 ± 0.8	9.9 ± 0.7	7.2 ± 1.1
Insulin (pmol/L)	61 ± 12	24 ± 6 *	8 ± 4***
NEFA (µmol/L)	528 ± 43	700 ± 87	263 ± 35*
Triglycerides (mmol/L)	0.58 ± 0.06	0.34 ± 0.02**	0.30 ± 0.04***
Leptin (ng/mL)	1.40 ± 0.42	1.09 ± 0.20	0.06 ± 0.06**

D

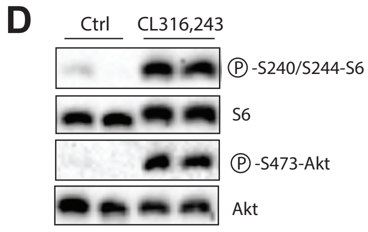

E. F. G. H.

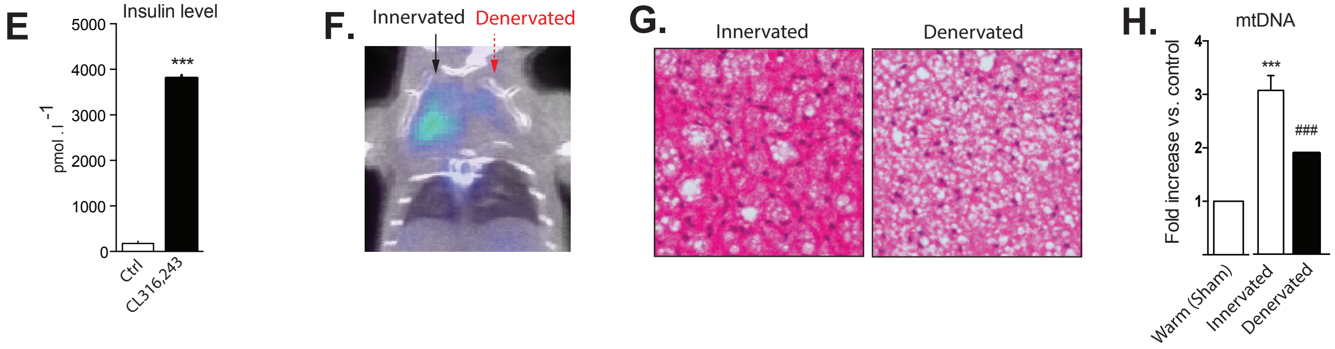

Figure S2

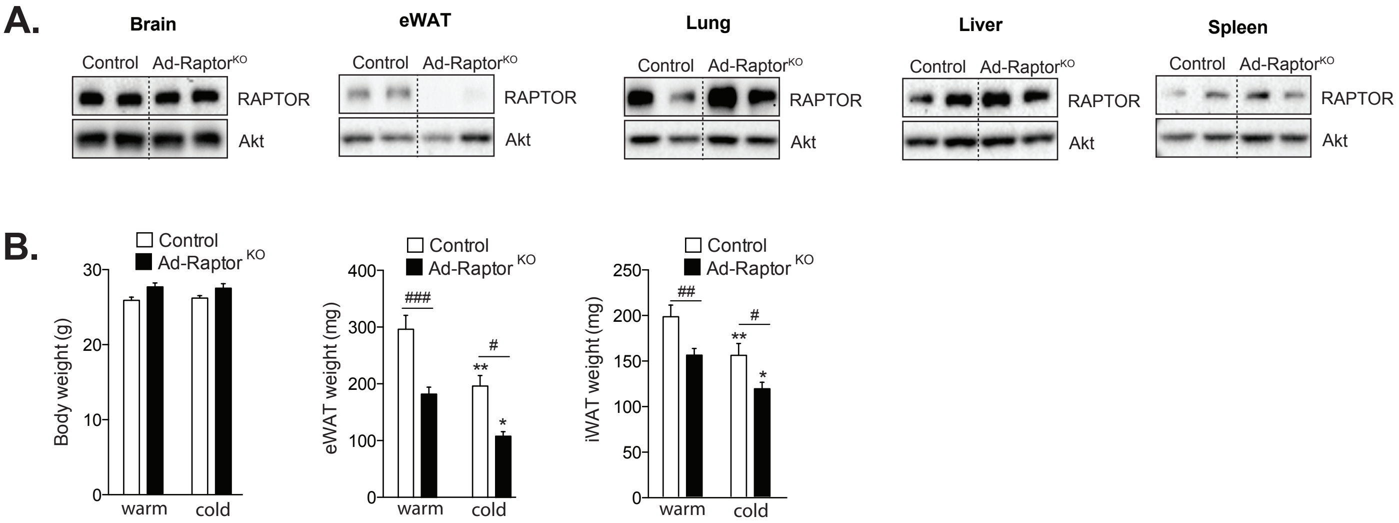

C.

	Control			Ad-Raptor^{KO}		
	warm	cold		warm	cold	
		acute	chronic		acute	chronic
Glucose (mmol/L)	9.7 ± 0.5	9.9 ± 0.8	10.3 ± 0.9	10.4 ± 0.5	9.9 ± 0.9	6.4 ± 0.5$^{\&\&\&}_{\#\#\#}$
Insulin (pmol/L)	49 ± 10	14 ±4	7 ± 3*	136 ± 36$^{\#\#\#}$	14 ± 4$^{\&\&\&}$	18 ± 7$^{\&\&\&}$
NEFA (μmol/L)	608 ± 76	863 ± 81*	436 ± 14	364 ± 18$^{\#}$	835 ± 81$^{\&\&\&}$	294 ± 32
Triglycerides (mmol/L)	0.52 ± 0.03	0.41 ± 0.02	0.31 ± 0.03**	0.43 ± 0.07	0.34 ± 0.02	0.40 ± 0.04
Leptin (ng/mL)	1.53 ± 0.32	0.93 ± 0.18*	0.11 ± 0.04***	0.44 ± 0.11$^{\#\#\#}$	0.49 ± 0.17	0.12 ± 0.05$^{\&\&}$

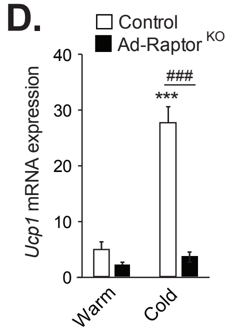

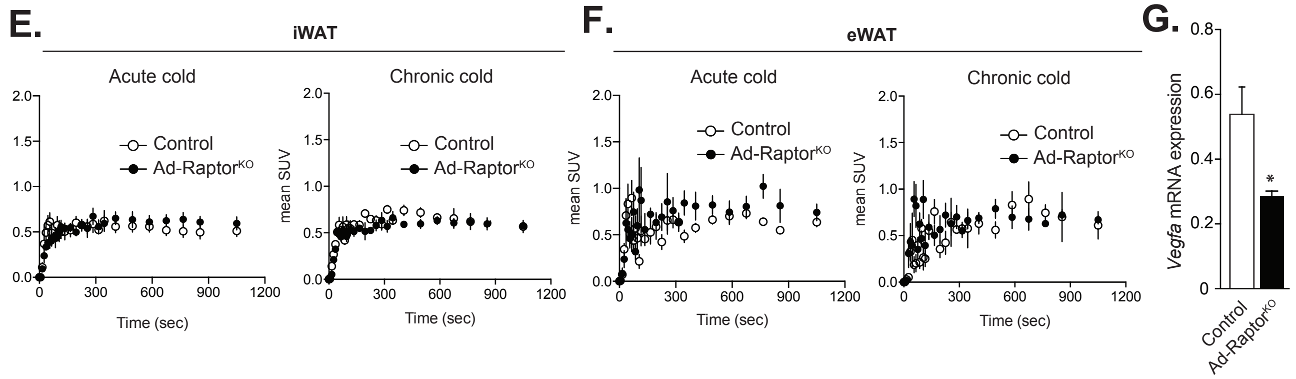

Figure S3

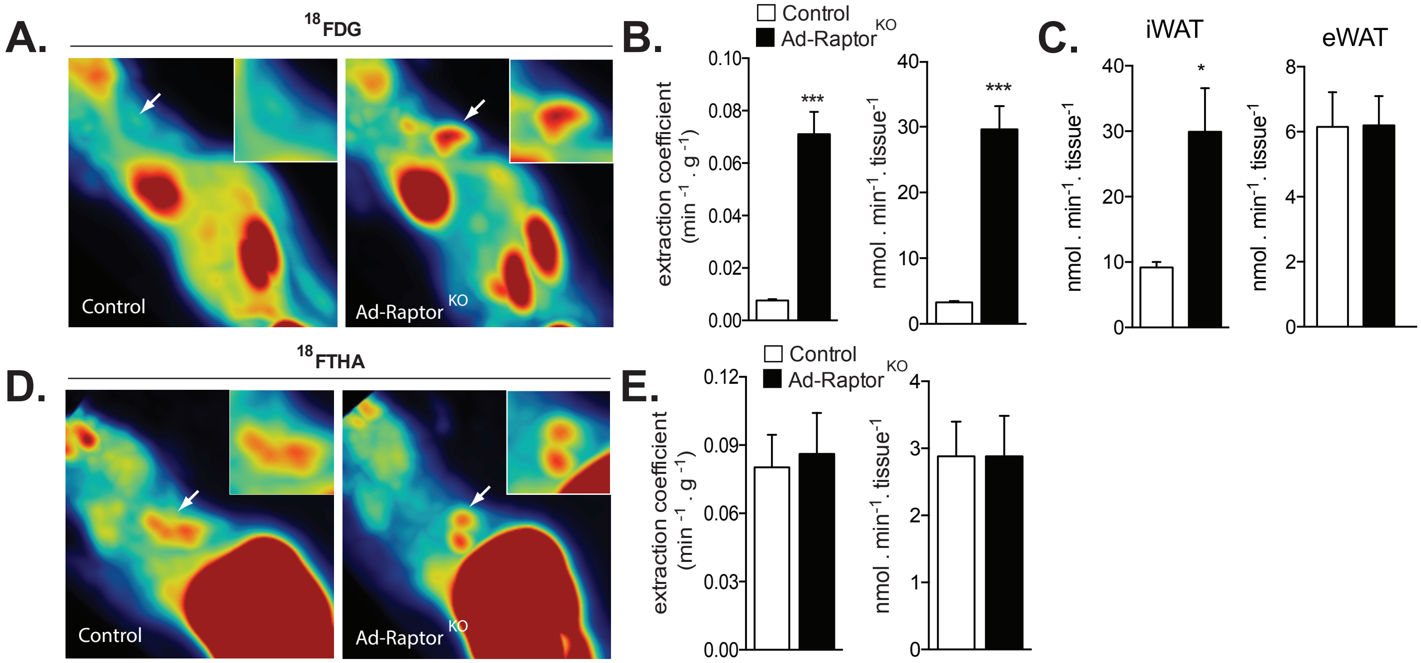
A.
^{18}FDG
Control
Ad-Raptor KO
B.
Control
Ad-Raptor KO
extraction coefficient (min -1 . g -1)
nmol . min-1. tissue-1
***
C.
iWAT
eWAT
nmol . min-1. tissue-1
*
D.
^{18}FTHA
Control
Ad-Raptor KO
E.
Control
Ad-Raptor KO
extraction coefficient (min -1 . g -1)
nmol . min-1. tissue-1

Figure S4

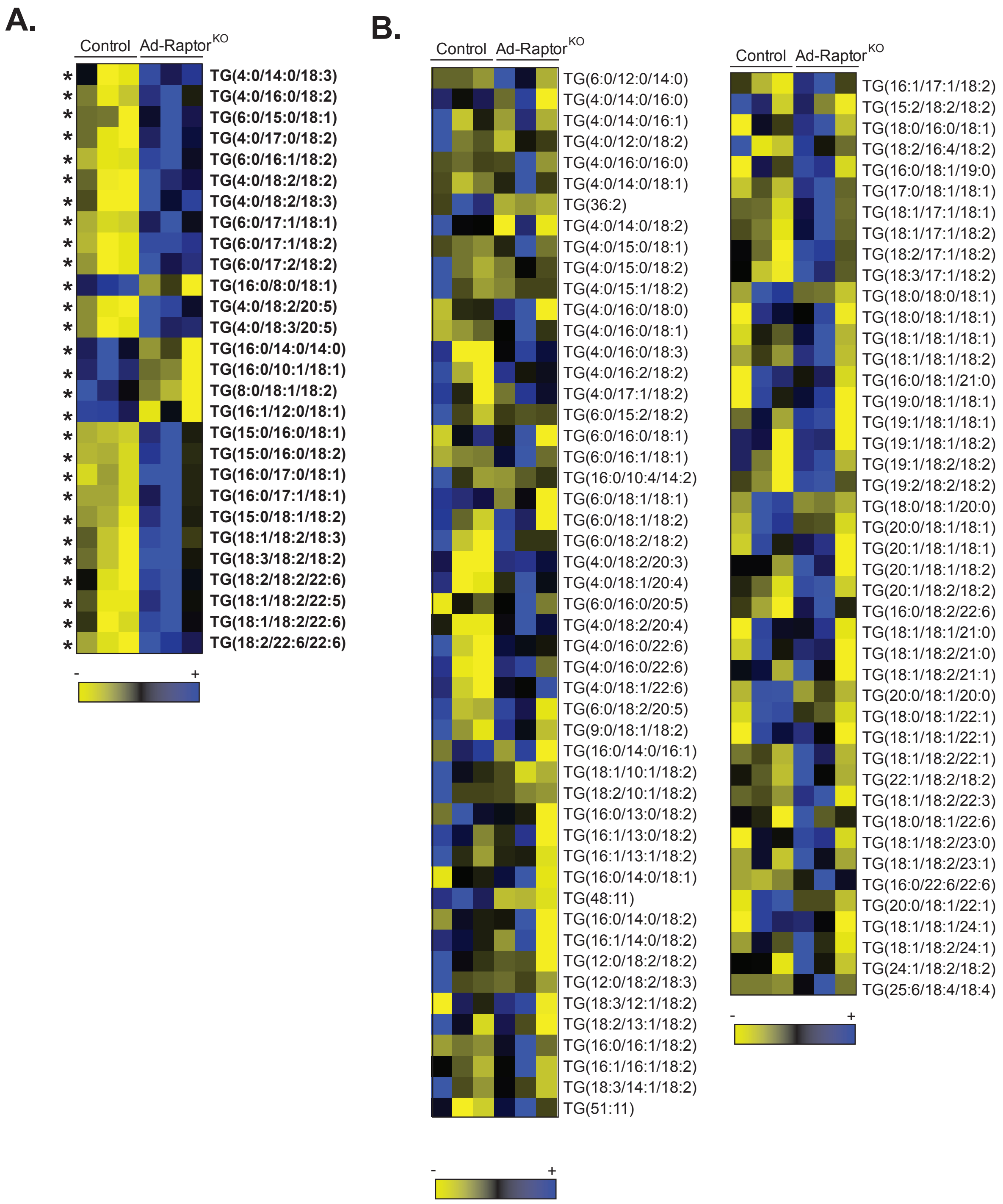
A.
Control
Ad-Raptor KO
* TG(4:0/14:0/18:3)
* TG(4:0/16:0/18:2)
* TG(6:0/15:0/18:1)
* TG(4:0/17:0/18:2)
* TG(6:0/16:1/18:2)
* TG(4:0/18:2/18:2)
* TG(4:0/18:2/18:3)
* TG(6:0/17:1/18:1)
* TG(6:0/17:1/18:2)
* TG(6:0/17:2/18:2)
* TG(16:0/8:0/18:1)
* TG(4:0/18:2/20:5)
* TG(4:0/18:3/20:5)
* TG(16:0/14:0/14:0)
* TG(16:0/10:1/18:1)
* TG(8:0/18:1/18:2)
* TG(16:1/12:0/18:1)
* TG(15:0/16:0/18:1)
* TG(15:0/16:0/18:2)
* TG(16:0/17:0/18:1)
* TG(16:0/17:1/18:1)
* TG(15:0/18:1/18:2)
* TG(18:1/18:2/18:3)
* TG(18:3/18:2/18:2)
* TG(18:2/18:2/22:6)
* TG(18:1/18:2/22:5)
* TG(18:1/18:2/22:6)
* TG(18:2/22:6/22:6)
-
+
B.
Control
Ad-Raptor KO
TG(6:0/12:0/14:0)
TG(4:0/14:0/16:0)
TG(4:0/14:0/16:1)
TG(4:0/12:0/18:2)
TG(4:0/16:0/16:0)
TG(4:0/14:0/18:1)
TG(36:2)
TG(4:0/14:0/18:2)
TG(4:0/15:0/18:1)
TG(4:0/15:0/18:2)
TG(4:0/15:1/18:2)
TG(4:0/16:0/18:0)
TG(4:0/16:0/18:1)
TG(4:0/16:0/18:3)
TG(4:0/16:2/18:2)
TG(4:0/17:1/18:2)
TG(6:0/15:2/18:2)
TG(6:0/16:0/18:1)
TG(6:0/16:1/18:1)
TG(16:0/10:4/14:2)
TG(6:0/18:1/18:1)
TG(6:0/18:1/18:2)
TG(6:0/18:2/18:2)
TG(4:0/18:2/20:3)
TG(4:0/18:1/20:4)
TG(6:0/16:0/20:5)
TG(4:0/18:2/20:4)
TG(4:0/16:0/22:6)
TG(4:0/16:0/22:6)
TG(4:0/18:1/22:6)
TG(6:0/18:2/20:5)
TG(9:0/18:1/18:2)
TG(16:0/14:0/16:1)
TG(18:1/10:1/18:2)
TG(18:2/10:1/18:2)
TG(16:0/13:0/18:2)
TG(16:1/13:0/18:2)
TG(16:1/13:1/18:2)
TG(16:0/14:0/18:1)
TG(48:11)
TG(16:0/14:0/18:2)
TG(16:1/14:0/18:2)
TG(12:0/18:2/18:2)
TG(12:0/18:2/18:3)
TG(18:3/12:1/18:2)
TG(18:2/13:1/18:2)
TG(16:0/16:1/18:2)
TG(16:1/16:1/18:2)
TG(18:3/14:1/18:2)
TG(51:11)
-
+
Control
Ad-Raptor KO
TG(16:1/17:1/18:2)
TG(15:2/18:2/18:2)
TG(18:0/16:0/18:1)
TG(18:2/16:4/18:2)
TG(16:0/18:1/19:0)
TG(17:0/18:1/18:1)
TG(18:1/17:1/18:1)
TG(18:1/17:1/18:2)
TG(18:2/17:1/18:2)
TG(18:3/17:1/18:2)
TG(18:0/18:0/18:1)
TG(18:0/18:1/18:1)
TG(18:1/18:1/18:1)
TG(18:1/18:1/18:2)
TG(16:0/18:1/21:0)
TG(19:0/18:1/18:1)
TG(19:1/18:1/18:1)
TG(19:1/18:1/18:2)
TG(19:1/18:2/18:2)
TG(19:2/18:2/18:2)
TG(18:0/18:1/20:0)
TG(20:0/18:1/18:1)
TG(20:1/18:1/18:1)
TG(20:1/18:1/18:2)
TG(20:1/18:2/18:2)
TG(16:0/18:2/22:6)
TG(18:1/18:1/21:0)
TG(18:1/18:2/21:0)
TG(18:1/18:2/21:1)
TG(20:0/18:1/20:0)
TG(18:0/18:1/22:1)
TG(18:1/18:1/22:1)
TG(18:1/18:2/22:1)
TG(22:1/18:2/18:2)
TG(18:1/18:2/22:3)
TG(18:0/18:1/22:6)
TG(18:1/18:2/23:0)
TG(18:1/18:2/23:1)
TG(16:0/22:6/22:6)
TG(20:0/18:1/22:1)
TG(18:1/18:1/24:1)
TG(18:1/18:2/24:1)
TG(24:1/18:2/18:2)
TG(25:6/18:4/18:4)
-
+

Figure S5

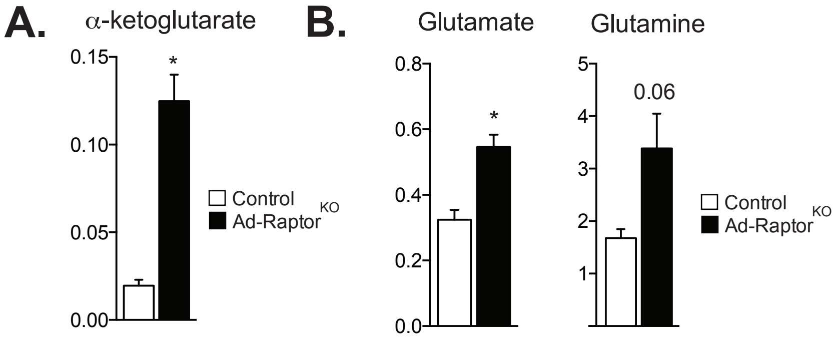
A.
α-ketoglutarate
0.15
0.10
0.05
0.00
*
Control
Ad-Raptor KO
B.
Glutamate
0.8
0.6
0.4
0.2
0.0
*
Glutamine
5
4
3
2
1
0.06
Control
Ad-Raptor KO

Figure S6

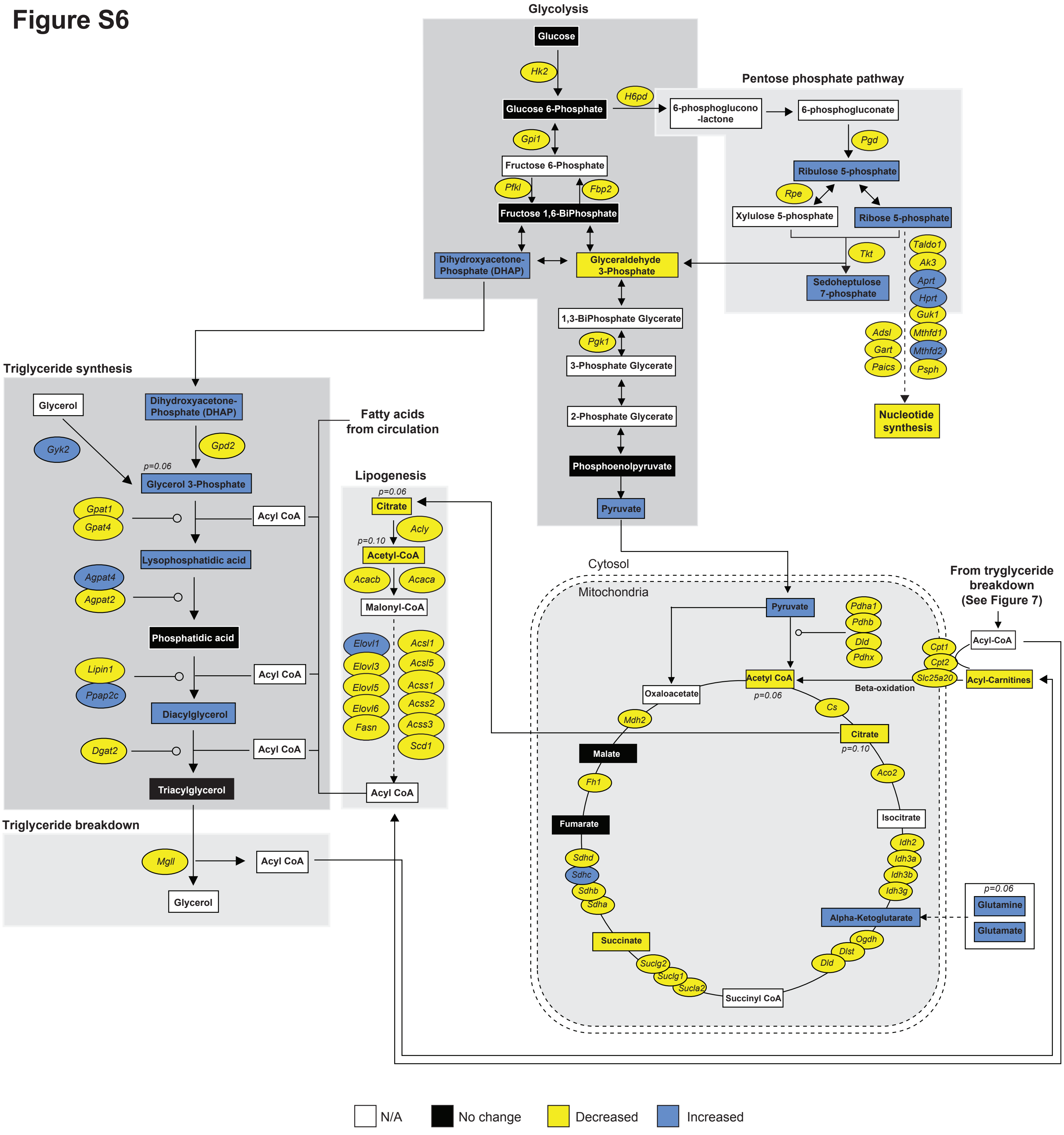

Glycolysis
Glucose
Hk2
Glucose 6-Phosphate
H6pd
Gpi1
Fructose 6-Phosphate
Pfkl
Fbp2
Fructose 1,6-BiPhosphate
Dihydroxyacetone-Phosphate (DHAP)
Glyceraldehyde 3-Phosphate
1,3-BiPhosphate Glycerate
Pgk1
3-Phosphate Glycerate
2-Phosphate Glycerate
Phosphoenolpyruvate
Pyruvate
Pentose phosphate pathway
6-phosphoglucono -lactone
6-phosphogluconate
Pgd
Ribulose 5-phosphate
Rpe
Xylulose 5-phosphate
Ribose 5-phosphate
Tkt
Sedoheptulose 7-phosphate
Taldo1
Ak3
Aprt
Hprt
Guk1
Mthfd1
Mthfd2
Psph
Adsl
Gart
Paics
Nucleotide synthesis
Triglyceride synthesis
Glycerol
Dihydroxyacetone-Phosphate (DHAP)
Gyk2
Gpd2
p=0.06
Glycerol 3-Phosphate
Gpat1
Gpat4
Acyl CoA
Lysophosphatidic acid
Agpat4
Agpat2
Phosphatidic acid
Lipin1
Ppap2c
Acyl CoA
Diacylglycerol
Dgat2
Acyl CoA
Triacylglycerol
Fatty acids from circulation
Lipogenesis
p=0.06
Citrate
Acly
p=0.10
Acetyl-CoA
Acacb
Acaca
Malonyl-CoA
Elovl1
Elovl3
Elovl5
Elovl6
Fasn
Acsl1
Acsl5
Acss1
Acss2
Acss3
Scd1
Acyl CoA
Triglyceride breakdown
Mgll
Acyl CoA
Glycerol
Cytosol
Mitochondria
Pyruvate
Pdha1
Pdhb
Dld
Pdhx
Oxaloacetate
Acetyl CoA
p=0.06
Cs
Beta-oxidation
Mdh2
Citrate
p=0.10
Malate
Fh1
Aco2
Fumarate
Isocitrate
Sdhd
Sdhc
Sdhb
Sdha
Idh2
Idh3a
Idh3b
Idh3g
Alpha-Ketoglutarate
Succinate
Ogdh
Dlst
Dld
Suclg2
Suclg1
Sucla2
Succinyl CoA
From tryglyceride breakdown (See Figure 7)
Acyl-CoA
Cpt1
Cpt2
Slc25a20
Acyl-Carnitines
p=0.06
Glutamine
Glutamate
N/A
No change
Decreased
Increased

Figure S7

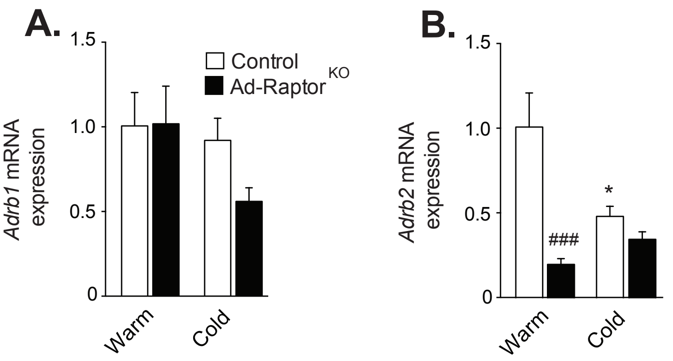
A.
1.5
1.0
0.5
0
Adrb1 mRNA expression
Control
Ad-Raptor KO
Warm
Cold
B.
1.5
1.0
0.5
0
Adrb2 mRNA expression
###
*
Warm
Cold
